# Abstracts from Hydrocephalus 2021: The Thirteenth Meeting of the International Society for Hydrocephalus and Cerebrospinal Fluid Disorders

**DOI:** 10.1186/s12987-021-00293-w

**Published:** 2021-12-21

## Abstracts: Young Investigators

### Y01 A multivariate analysis of 82 consecutive patients shunted for idiopathic normal pressure hydrocephalus

#### Amit R Persad^1^, Bashir Daud Shah^2^, Kotoo Meguro^1^

##### ^1^Division of Neurosurgery, Department of Surgery, University of Saskatchewan, Canada; ^2^Division of Neurology, Department of Medicine, University of Saskatchewan, Canada

###### **Correspondence:** Amit Persad (amit.persad@usask.ca)

*Fluids Barriers CNS* 2021, **18(2)**: Y01

**Introduction:** Idiopathic normal pressure hydrocephalus (iNPH) can be treated with shunting. Little evidence exists to guide shunt selection, predictors of success, or patient follow- up.

**Methods:** We performed retrospective reveiw of 82 consecutive iNPH patinets treated with shunting for iNPH between 2007 and 2018. Clinical factors included age, sex, Charlson Comorbidity Index (CCI), presence of hypertension and diabetes and follow-up. Surgical factors included pre-op spinal tap, type of shunt (LP, VP, fixed, adjustable), use of laparoscopic assistance and having surgery done by hydrocephalus specialist surgeon. Imaging factors included callosal angle (CA) and disproportional enlargement subarachnoid space hydrocephalus (DESH). Regressional statistics were performed.

**Results:** 52 male and 30 female, patients were identified with average age 71.4 years. The cohort mRS improved from 3.84 to 2.66 post-operatively (p < 0.005). 63.6% of patients had clinical improvement with shunt surgery in the short-term and 48.7% in the long-term. Factors that predicted better shunt outcome short-term were lower CCI ( < 0.05), absence of hypertension ( < 0.05), more intensive follow-up (< 0.05) and pre-op CA ≤ 80° (<0.05). Factors that predicted better shunt outcome long-term were younger age at surgery (< 0.05), use of laparoscopic approach (< 0.005) and pre-op DESH (< 0.05). Factors that predicted reduced complications were smaller pre-op CA (< 0.05), use of laparoscopic approach (< 0.05), utilization of LP shunt (< 0.05) and having surgery done by hydrocephalus specialist (< 0.05).

**Conclusion:** In our centre, iNPH patients had improvement following shunting. Age, CCI, CA, DESH, more intensive follow-up, absence of hypertension and use of laparoscopic approach helped predict success of shunting in iNPH patients.

### Y02 A novel model of acquired hydrocephalus for evaluation of neurosurgical treatments

#### Sarah Zwick^1^, Pat McAllister^1^, Michael Talcott^1,2^, Albert Isaacs^3^, Maria Garcia-Bonilla^1^, Leandro Castaneyra-Ruiz^1^, Alexis Hartman^1^, Ryan Dilger^4,5^, Stephen Fleming^4,5^, Rebecca Golden^4^, Diego Morales^1^, Carolyn Harris^6,7^, David Limbrick Jr.^1,8^

##### ^1^Department of Neurosurgery, Washington University in St. Louis School of Medicine, St. Louis, Missouri, 63110, USA; ^2^Division of Comparative Medicine, Washington University in St. Louis School of Medicine, St. Louis, Missouri, 63110, USA; ^3^Department of Surgery, Division of Neurosurgery, University of Calgary School of Medicine, Calgary, Alberta, T2N2T0, Canada; ^4^Department of Animal Sciences, Division of Nutritional Sciences, Neuroscience Program, University of Illinois, Champaign-Urbana, Illinois, 61801, USA; ^5^Traverse Science, Champaign, Illinois, 61801, USA; ^6^Department of Chemical Engineering and Materials Science, Wayne State University, Detroit, Michigan, 48202, USA; ^7^Department of Neurosurgery, Wayne State University School of Medicine, Detroit, Michigan, 48202, USA; ^8^Department of Pediatrics, St. Louis Children’s Hospital, St. Louis, Missouri, 63110, USA

###### **Correspondence:** Sarah Zwick (sarahzwick@hotmail.com)

*Fluids Barriers CNS* 2021, **18(2)**: Y02

**Introduction:** Many animal models have been used to study the pathophysiology of hydrocephalus; most have been rodent models whose lissencephalic cerebral cortex may respond to ventriculomegaly differently than gyrencephalic brains, and whose size is not amenable to clinically relevant neurosurgical treatments. Thus, we present a porcine model of hydrocephalus in juvenile pigs and established shunting and endoscopic treatment methods.

**Methods:** Acquired hydrocephalus was induced in 33-39-day old pigs by percutaneous intracisternal kaolin injections (n = 32). Controls received saline-injection (sham, n = 6) or none (intact, n = 4). MRI evaluated the progression of ventriculomegaly prior to surgery. With the aid of Stealth neuronavigation, ventriculoperitoneal shunts were inserted (n = 10) or choroid plexus cauterization (CPC, n = 3), with or without endoscopic third ventriculostomy (ETV+CPC, n = 5), were performed 1-4 weeks post-kaolin. Neurological status was assessed daily, and novel- object recognition tests (NOR) assessed cognition.

**Results:** Bilateral ventriculomegaly in all cerebral ventricles occurred post-induction from solid casts of kaolin in the basal cisterns with a patent cisterna magna. In 17 untreated hydrocephalic animals, mean total ventricular volumes were 8158 + 5466 SD mm^3^, significantly larger than control values of 2251 + 213 SD mm^3^ (p < 0.0005). Although untreated pigs were asymptomatic despite exhibiting chronic moderate-severe ventriculomegaly, NOR testing revealed cognitive changes after kaolin injection. Treated animals developed ataxia and lethargy in the setting of shunt or ETV-CPC failure.

**Conclusion:** Mechanical induction of acquired hydrocephalus produces a useful, translational in vivo model that allows systematic studies of the pathophysiology and clinical treatment of hydrocephalus.

### Y03 Biochemical profile of human infant cerebrospinal fluid in intraventricular hemorrhage and post-hemorrhagic hydrocephalus of prematurity

#### Ayodamola Otun^1^, Diego Morales^1,2^, James P. McAllister II^1^, Maria Garcia-Bonilla^1^, David Limbrick^1,2^

##### ^1^Department of Pediatric Neurosurgery, Washington University, St. Louis, MO, 63110, USA; ^2^St. Louis Children’s Hospital, MO, 63110, USA

###### **Correspondence:** Ayodamola Otun (aotun@wustl.edu)

*Fluids Barriers CNS* 2021, **18(2)**: Y03

**Introduction:** Intraventricular hemorrhage (IVH) and post-hemorrhagic hydrocephalus (PHH) have complex pathophysiology involving cell-junction disruption, and choroid-plexus (ChP) hypersecretion. Increased CSF cytokines, matrix proteins, and blood metabolites occur in IVH/PHH, and increased CSF electrolytes/osmolality have been shown in hydrocephalus but not in IVH/PHH. We hypothesized that total protein, osmolality, and electrolytes in CSF increase in PHH.

**Methods:** CSF osmolality, total protein, and electrolytes were measured in 69 infants (26 controls, 18 IVH I/II, 14 IVH III/IV, and 11 PHH). Serum electrolyte concentrations within 1-day of clinical sampling were obtained from clinical charts.

**Results:** CSF osmolality, sodium, potassium, chloride, and magnesium were increased in PHH compared to control, IVH I/II, and IVH III/IV (p ≤ 0.0001–0.03). CSF total protein was increased in IVH III/IV and PHH compared to control and IVH I/II (p ≤ 0.0001-0.03). CSF bicarbonate and calcium were increased in IVH III/IV and PHH compared to IVH I/II (p = 0.0004-0.02). Quantitatively, 87% of CSF osmolality change between control and PHH are due to sodium and chloride concentration changes. Serum osmolality, total protein, and electrolytes except calcium were not changed between all groups.

**Conclusion:** CSF osmolality, and sodium, potassium, chloride, and magnesium levels increased only in PHH. However, total protein increased in both IVH III/IV and PHH. Serum electrolytes levels were unchanged across groups and lower than CSF values suggesting increased CSF electrolytes may be more likely secondary to increased ChP ion secretion rather than blood-CSF-barrier disruption. Findings also suggest osmolality changes may be due to electrolyte changes and might contribute to the development of PHH.

### Y04 Change in CSF pocket size after shunt placement in normal pressure hydrocephalus

#### Emanuele Camerucci^1^, Jonathan Graff-Radford^2^, David T. Jones^2^, Benjamin D. Elder^3,4^, Jeffrey L. Gunter^1^, Jeremy Cutsforth-Gregory^2^, Hugo Botha^2^, Matthew C. Murphy^1^, Clifford, R. Jack Jr^1^, John Huston III^1^, Petrice M. Cogswell^1^

##### ^1^Department of Radiology, Mayo Clinic, Rochester, MN, 55905, USA; ^2^Department of Neurology, Mayo Clinic, Rochester, MN 55905, USA; ^3^Department of Neurologic Surgery, Mayo Clinic, Rochester, MN 55905, USA; ^4^Department of Physiology and Biomedical Engineering, Mayo Clinic, Rochester, MN 55905, USA

###### **Correspondence:** Emanuele Camerucci (camerucci.emanuele@mayo.edu)

*Fluids Barriers CNS* 2021, **18(2)**: Y04

**Introduction:** Cerebrospinal fluid (CSF) pockets are a component of disproportionately enlarged subarachnoid space hydrocephalus (DESH), used in some diagnostic criteria for idiopathic normal pressure hydrocephalus (iNPH). CSF pockets may be mistaken for brain atrophy. The purpose of this study is to assess for change in CSF pocket size after shunt placement in patients with iNPH.

**Methods:** Inclusion criteria were clinical diagnosis of iNPH, ventriculoperitoneal shunt placement, and at least one MRI before and after shunt placement, performed from January 2015 through March 2021. We evaluated for the presence of CSF pockets on the pre-shunt MRI. For each pocket, the volume was estimated as an ellipsoid by measuring the x, y, and z dimensions on the pre-shunt MRI and each post-shunt MRI. A right-sided Wilcoxon signed rank test was performed to assess for decrease in size between the pre-shunt and first post-shunt MRI and similarly between the first post-shunt and last post-shunt MRI.

**Results:** CSF pockets were present in 54/137 (39%) patients. CSF pocket volume decreased by 19.0% (median 1,630mm^3^ [Q1:371; Q3:6,701], p < 0.001) between the pre-shunt and first post- shunt MRI and by 16.9% (1,189mm^3^ [Q1:55; Q3:3,200], p < 0.001) between the first and the last post-shunt MRI at median 13.1 months post-shunt (Q1:8.1; Q3:18.5).

**Conclusion:** CSF pockets decrease in size after shunt placement in patients with iNPH and continue to decrease in size in the following months. Findings provide further support for CSF pockets as an indicator of disordered CSF dynamics, and these pockets should be differentiated from atrophy.

### Y05 Establishment of age and gender specific normal cerebral ventricle volumes

#### Harrison Synder^1^, Xue Feng^2^, Min Park^3^, Jan Vargas^4^, Ryan T Kellogg^3^

##### ^1^School of Medicine, University of Virginia, Charlottesville, VA, 22901, USA; ^2^Department of Biomedical Engineeering, University of Virginia, Charlottesville, VA, 22901, USA; ^3^Department of Neurological Surgery, University of Virginia, Charlottesville, VA, 22901, USA; ^4^Division of Neurosurgery, Prisma Health, Greenville, SC, 29605, USA

###### **Correspondence:**  Ryan T Kellogg (rtk4u@virginia.edu)

*Fluids Barriers CNS* 2021, **18(2)**: Y05

**Introduction:** Ventriculomegaly is the most utilized radiographic finding of hydrocephalus. To date there has been no major reports of normative ventricle size for the adult population. Establishing a normative dataset would provide an objective volumetric measurement to help guide diagnosis of hydrocephalus. Our goal was to generate a normative data set of ventricular volume utilizing non pathologic CT scans for adults.

**Methods:** The authors performed a retrospective analysis of non-contrast head CTs for adults that did not have a diagnosis of hydrocephalus or history of VP shunting or treatments for hydrocephalus. A convolutional neural network was trained on hand segmented scans from a variety of age ranges and then utilized to automate the segmentation of the entire data set. Ventricular volumes were produced for 1159 CT scans to generate a normative database.

**Results:** A convolutional neural network was trained using 201 scans and achieved a Dice score of 0.91±0.05 on 16 validation scans. This network generated ventricular volumes on 1159 CT scans. The ventricle volumes were binned by age ranges (18-29, 30-39, 40-49, 50-59, 60-69, 70-79, 80-89, and >90 years) and significantly larger sizes were observed for older ages (e.g. male 80-89: 73,095±6,263 mm^3^, 51-60: 31,486±12,732 mm^3^).

**Conclusion:** We have developed a convolutional neural network that can segment the ventricles on CT scans of adult patients for all ages. Using this entwork, a normative database that could be utilized in the future to help aid in the diagnosis of hydrocephalus based on normal ventricular volumes was generated.

### Y06 Hydrocephalus in paediatric posterior fossa tumors: the role of pre-operative etv and analysis of risk factors for CSF diversion following tumour resection

#### Lucia Darie, Richard D.C. Moon, Michael R. Carter, Greg A. Fellows, Richard J. Edwards

##### ^1^Department of Paediatric Neurosurgery, Bristol Royal Hospital for Children, University Hospitals Bristol and Weston NHS Foundation Trust, Bristol, UK

###### **Correspondence:** Lucia Darie (darielucia@yahoo.com)

*Fluids Barriers CNS* 2021, **18(2)**: Y06

**Introduction:** This study aimed to identify radiological markers and validate existing prediction rules that predicted the need for CSF diversion following posterior fossa tumour resection.

**Methods:** Cases were identified from a prospectively maintained database (2015- 2020). Clinical records were reviewed retrospectively. The modified Canadian preoperative prediction rule for hydrocephalus score (mCPPRH) was applied. Imaging review assessed the following: fronto-occipital horn ratio (FOHR), optic nerve sheet diameter (ONSD); brain, ventricular, and transependymal oedema volumes were determined using 3D segmentation software.

**Results:** 63 children with a new cerebellar/fourth ventricle tumour (34 pilocytic astrocytoma, 19 medulloblastoma, 6 ependymoma, 4 miscellaneous) were identified. Eighteen (29%) required no pre-operative CSF diversion; 13/18 (72%) of these were pilocytic astrocytoma; the mean values are: ONSD(right) 4.6mm (S D±1.18 mm), FOHR 0.35 (SD ±0.04), mCPPRH 2 (SD±2.5); ventricles occupied 6.2% of the intracranial volume. Forty-two children (67%) had a pre- resection endoscopic third ventriculostomy (ETV). Images were available for 37 ETV patients; the mean values are: ONSD(right) 6.54mm (SD ±0.95), FOHR 0.44(SD ±0.5), mCPPRH 4.2 (SD ±1.5); ventricles and transependymal oedema occupied 17.8% and 3.5% of the intracranial volume, respectively. The values in the non-intervention vs ETV groups were significantly different (p ≤0.05). 9/42 (21%) failed ETV requiring a ventriculoperitoneal shunt; these patients had more severe hydrocephalus and higher mCPPRH scores: FOHR 0.48 (SD ±0.06), mCPPRH 5.2 (SD ±1.7).

**Conclusion:** The risk of hydrocephalus correlates with tumor etiology. ETV failed in children with higher FOHR and mCPPRH scores. The mCPPRH is validated in this international patient cohort.

**Keywords**: Paediatric posterior fossa tumors, Hydrocephalus, Endoscopic third ventriculostomy, Ventriculoperitoneal shunt, Papilledema, Optic nerve sheet diameter, FOHR, Modified Canadian preoperative prediction rule for hydrocephalus score (mCPPRH), Surface area calculation, 3D Slicer

### Y07 Initial experiences with miethke m.blue^®^ valve in inph patients

#### Petr Skalický^1,3^, Arnošt Mládek^1,2^, Aleš Vlasák^3^, Helen Whitley^3^, Ondřej Bradáč^1,3^

##### ^1^Department of Neurosurgery and Neurooncology, 1st Faculty of Medicine, Charles University and Military University Hospital, Prague, Czech Republic; ^2^Department of Cognitive Systems and Neurosciences, Czech Institute of Informatics, Robotics and Cybernetics, Czech Technical University, Prague, Czech Republic; ^3^Department of Neurosurgery, 2nd Faculty of Medicine, Charles University and Motol University Hospital, Prague, Czech Republic

###### **Correspondence:** Petr Skalicky (skalicky.petr@uvn.cz)

*Fluids Barriers CNS* 2021, **18(2)**: Y07

**Introduction:** Adjustable valves and devices preventing the so-called siphon effect received the greatest support in the literature for hydrocephalus treatment. This work describes the first experiences with an adjustable gravitational valve with fixed differential pressure unit M.blue® in iNPH patients

**Methods:** 21 patients were indicated to shunt surgery based on clinical symptoms, radiological sings, LIT results and improvement of the symptoms after ELD mimicking probable iNPH diagnosis according to the Japanese guidelines. Neuropsychological testing battery, Dutch Gait Scale, ICIQ-UI SF, SF12V2-Health Survey, Kiefer Scale and 3T MRI were used to measure outcomes at 3-month control. Angle of the valve, BMI, etc. were studied for any relation to the risk of overdrainage. Valves were set according to the manufacturer's recommendations.

**Results:** Significant improvement at 3months was seen in Kiefer Scale, ICIQ-UI SF, MCS-12 (SF12), Dutch Gait Scale. Neuropsychological testing battery remained stable. 7 patients needed more than one adjustment of the valve. This subgroup significantly improved only in Dutch Gait Scale and MCS-12 but the trend was toward significant improvement in other variables. 8 patients had subdural effusions that were completely managed with adjustments until the 3-month control. BMI was significantly lower in patients with ≥2 adjustments than those with a maximum of one adjustment.

**Conclusion:** The initial results with M.blue® shunt system in iNPH patients are promising. More studies are needed to provide rationale for its use in iNPH. We recommend increasing the initial valve setting by 2-4 cm H2O, especially in lean patient.

### Y08 Periventricular white matter changes are associated with less improvement after shunt surgery in iNPH patients

#### Carl Snöbohm^1^, Filip Malmberg^2^, David Fällmar^3^, Johan Virhammar^1^

##### ^1^Department of Neuroscience, Neurology, Uppsala University, Sweden; ^2^Department of Information Technology, Division of Visual Information and Interaction, Uppsala University, Sweden; ^3^Department of Surgical Sciences, Radiology, Uppsala University, Sweden

###### **Correspondence:** Carl Snobohm (car.snobohm@gmail.com)

*Fluids Barriers CNS* 2021, **18(2)**: Y08

**Introduction:** Hyperintense white matter changes (WMC) on brain imaging can be classified as deep white matter hyperintensities (DWMH) or periventricular hyperintensities (PVH) and are frequently seen in patients with idiopathic normal pressure hydrocephalus (iNPH). Contradictory results have been reported as to whether preoperative WMC are associated with outcome after shunt surgery in iNPH patients. The aim was to investigate the predictive role of PVH and DWMH on shunt outcome in iNPH patients using magnetic resonance (MR) volumetry.

**Methods:** A total of 253 iNPH patients were included that were operated with shunt surgery between 2011 and 2015 and clinically assessed before and 12 months after surgery. All patients were investigated preoperatively with an MRI of the brain. Volume of DWMH and PVH were quantified on fluid-attenuated inversion recovery images using an in house semi-automatic volumetric segmentation software (SmartPaint). Clinical outcome was defined as the difference in symptom score between post- and preoperative investigations, measured with the iNPH- scale.

**Results:** In a linear regression model, volume of PVH was negatively associated with shunt outcome (B = − 0.095, p = 0.042) after controlling for age and preoperative symptom score. Volume of DWMH was not associated with shunt outcome (p = 0.16). Whole brain WMC volume was associated with more severe cognitive symptoms at baseline (B = − 0.23, p = 0.008). In patients with high volume of PVH (>40 milliliters) 45% of the patients improved after shunting.

**Conclusion:** The volume of PVH correlates with less favorable shunt outcome in iNPH patients. Patients with high volume of PVH may still benefit from shunt surgery.

### Y09 Usefulness of desh in predicting outcome of extended lumbar drainage and shunt insertion

#### Adam Nunn^1^, Melissa Werndle^2^, Joao Alves Rosa^2^, Kelly McManus^1^, Rebecca Hodnett^1^, Jack Wildman^1^, William Singleton^1^, Alex Mortimer^2^, Richard Edwards^1^

##### ^1^Department of Neurosurgery, Southmead Hospital, Bristol, UK; ^2^Department of Neuroradiology, Southmead Hospital, Bristol, UK.

###### **Correspondence:** Adam Nunn (adam.nunn@nbt.nhs.uk)

*Fluids Barriers CNS* 2021, **18(2)**: Y09

**Introduction:** Disproportionately enlarged subarachnoid spaces hydrocephalus (DESH) is a series of radiological signs that together have been proposed to predict shunt responsiveness in patients with possible/suspected idiopathic normal pressure hydrocephalus (iNPH).

**Methods:** Clinical notes of 741 consecutive new patients presenting to our service over a 15- year period were reviewed and patients included if they were felt to have possible/suspected iNPH and had no evidence of secondary NPH. The presence or absence of DESH was not considered when selecting patients for shunting. Clinical data was extracted at baseline, pre- and post-ELD and at 3 and 12 months post-shunt insertion. The referral scans were subjected to blinded review by a neuroradiologist.

**Results:** Data were complete for 415 patients undergoing ELD (49.2% DESH) and 323 patients undergoing shunt insertion (47.4% DESH). Complete agreement was seen between the neuroradiologists regarding the diagnosis of DESH during independent review of 15 scans. However, DESH did not predict objective improvement after ELD (likelihood ratio 0.15, P = 0.70), nor did its presence predict improvement at 3 or 12 months post-shunting (LR 1.7 [P = 0.20], LR 1.9 [P = 0.16], respectively). The magnitude of gait or cognitive improvement at 3 and 12 months was also not statistically significantly different.

**Conclusion:** In this large population of patients with possible/suspected iNPH (confirmed to have no evidence of decompensated arrested or secondary NPH both clinically and radiologically), DESH did not predict shunt responsiveness, suggesting that its usefulness as a prognostic marker should be re-evaluated.

### Y10 variants in SWI/SNF complex component smarcc1 lead to developmental hydrocephalus and other syndromic features

#### Amrita K. Singh^1^, Stephen Viviano^2^, Phan Q. Duy^1^, Jay Ma^3^, August Allocco^1^, Tyrone Despenza^1^, Sheng Chih Jin^3^, Engin Deniz^2^, Kristopher T. Kahle^1^

##### ^1^Department of Neurosurgery, Yale University, New Haven, Connecticut, United States; ^2^Department of Pediatrics, Yale University, New Haven, Connecticut, United States; ^3^Department of Genetics, Washington University School of Medicine, St Louis, Missouri, United States

###### **Correspondence:** Amrita Singh (amrita.singh@yale.edu)

*Fluids Barriers CNS* 2021, **18(2)**: Y010

**Introduction:** Congenital hydrocephalus (CH) is a disorder of ventricular expansion related to CSF physiology. CH has been linked to a number of genes including *SMARCC1*, a core SWI/ SNF complex subunit which regulates gene expression required for neural stem cell (NSC) proliferation during forebrain development. Recent whole exome sequencing studies have identified *SMARCC1* as a high confidence pathogenic gene in CH.

**Methods:** We collected a cohort of 12 CH patients with *SMARCC1* variants and used a Xenopus model to investigate observed phenotypes with optical coherence tomography, single cell RNA sequencing, in situ hybridization and immunohistochemistry.

**Results:** Aqueductal stenosis (AS) was present in all 12 patients, with cardiac and craniofacial defects each present in 3/12 patients. AS and ventriculomegaly were observed in the majority of *Smarcc1* Xenopus morphants and CRISPR knockdowns in both F0 and F1 generations. Importantly, we demonstrated rescue of the AS phenotype with human WT *SMARCC1* mRNA. Single cell RNA sequencing of human fetal tissue identified downregulation of NEUROD2, a gene related to NSC proliferation. In situ hybridization showed decreased expression of NEUROD2 in Xenopus morphants and knockdowns compared with WT in the prosencephalon. In contrast, surrounding the cerebral aqueduct, we found evidence of increased proliferation in *Smarcc1* morphants.

**Conclusion:** The clustering of phenotypes in *SMARCC1* human variants with replication in our Xenopus model illustrates variable expressivity and pleiotropy, suggesting that *SMARCC1* may define a novel Mendelian CH syndrome. Our results also provide mechanistic insight into pathogenesis of *SMARCC1*-related CH, which may enable identification of novel therapeutic targets.

### Y11 Ventricular volumetry for non-invasive evaluation of shunt function

#### Simon Lidén^1^, Dan Farahmand^2^, Katarina Laurell^1^

##### ^1^Department of Neuroscience, Neurology, Uppsala University, Sweden; ^2^Department of Clinical Neuroscience, Institute of Neuroscience and Physiology, The Sahlgrenska Academy, University of Gothenburg, and Department of Neurosurgery, Sahlgrenska university Hospital, Sweden

###### **Correspondence:** Simon Liden (simon.liden@hotmail.com)

*Fluids Barriers CNS* 2021, **18(2)**: Y011

**Introduction:** MRI-volumetry is an interesting alternative to invasive tests of shunt function. In this study we aimed to assess ventricular volume (VV) before and after surgery and at different opening pressure (OP) of the shunt.

**Methods:** The material consisted of 33 patients with a median (Md) age of 76 years with idiopathic normal pressure hydrocephalus who received a Strata® shunt with OP 1.5. Participants underwent MRI with volumetric sequences before surgery and four times postoperatively; at one month before randomization to either OP 1.0 or 2.5, at two months before crossover to OP 2.5 or 1.0; at three months before lowering to OP 0.5 and finally at three months and one day after surgery before resetting OP to 1.5. VV was measured semiautomatically using SyMRI®. Both the patient and the examiner were blinded to the OP.

**Results:** Significant changes were seen in VV from before (Md 129 ml) to one month after shunt surgery (Md 121 ml) and between OP 1.0 (Md 116 ml), 1.5 (Md 121 ml) and 2.5 (Md 127 ml) (p < 0.001). A unidirectional change in VV was seen for all participants between OP 1.0 and OP 2.5, (Md 11.5 ml, range 2.1-40.7) (p < 0.001). No significant change was seen in VV after 24 hours at OP 0.5.

**Conclusion:** The consistent decrease in VV after shunt surgery and between high and low OP of the shunt supports that MRI-volumetry could be a non-invasive method for evaluating shunt function, preventing unnecessary shunt revisions.

### Y12 Ventricular zone response to blood exposure at various time points

#### Mira Zaranek^1^, Rooshan Arshad^1^, Kevin Zheng^1^, Carolyn Harris PhD^1^

##### ^1^Chemical Engineering, Wayne State University, Detroit, Michigan, 48202, USA

###### **Correspondence:** Mira Zaranek (gf9749@wayne.edu)

*Fluids Barriers CNS* 2021, **18(2)**: Y012

**Introduction:** Posthemorrhagic hydrocephalus is the progressive dilation of the ventricular space following a hemorrhage within the brain. Exposure of the ventricular zone to blood is known to cause astrogliosis, microglial activation, cell junction dislocation, impairment of neural stem cell differentiation, and overall loss of ependymal cells, potentially leading to cerebrospinal fluid accumulation. In this study, the effect blood has on cellular response to a shunt catheter is evaluated.

**Methods:** Development of a novel custom-built 3D resin printed chamber was used to model the placement of a catheter sample on the ventricular zone. Undifferentiated neural stem cells extracted from C57BL/6 mice lateral ventricle and ATCC C8-D30 astrocytes were used. Cell counts were obtained to compared between control and whole blood exposed samples.

**Results:** Cells exposed to blood showed a significant increase (P < 0.0001) in astrocyte attachment when added concurrently with a catheter sample. The average total expression of DAPI on the sample exposed to blood was 392.0 ± 317.1 and 94.7 ± 44.5 for the control samples. Analysis of the GFAP stain expressed a total averages cell count of 174.3 ± 116.5 and 854.4 ± 450.7 for the sample not exposed to and exposed to blood, respectively.

**Conclusion:** An increase in cell count and a simultaneous increase in GFAP expression after whole blood exposure may be indicative of enhanced neuroinflammation, astrocyte activation, cytoskeletal changes, cell spreading, and/or increased cell communication. Altogether, these data suggest are indicative of the role blood products play in the activation of astrocytes and potentially shunt obstruction.

## Abstracts: Orals

### O01 Analysis of intracranial pressure pulse waveforms during infusion tests

#### Arkadiusz Ziółkowski^1^, Agata Pudełko^1^, Agnieszka Kazimierska^1^, Zofia Czosnyka^2^, Marek Czosnyka^2^, Magdalena Kasprowicz^1^

##### ^1^Department of Biomedical Engineering, Faculty of Fundamental Problems of Technology, Wrocław University of Science and Technology, Poland; ^2^Division of Neurosurgery, Department of Clinical Neurosciences, University of Cambridge, United Kingdom

###### **Correspondence:** Arkadiusz Ziolkowski (arkadiusz.ziolkowski@pwr.edu.pl)

*Fluids Barriers CNS* 2021, **18(2)**: O01

**Introduction:** The infusion test is often performed to select an appropriate treatment for hydrocephalus patients. It requires infusion of additional volume of normal saline into cerebrospinal fluid (CSF) space to calculate compensatory parameters. In this study we analysed the relationship between cerebrospinal elasticity and the intracranial pressure (ICP) pulse shape–derived parameters estimated before the external infusion was applied. We hypothesize that morphological analysis of ICP pulse waveforms at baseline may provide information on the state of CSF compensatory mechanisms.

**Methods:** Retrospective analysis of ICP and cerebral arterial blood flow velocity (CaBFV) recorded in 30 hydrocephalus patients (median age: 58) during infusion tests was performed. We calculated three ICP pulse shape–derived parameters: the ratio of the first and second peak of ICP pulse wave (P1/P2), the dominant morphological class of ICP pulse wave (on the scale from normal to pathological shape), and the ratio of pulse slopes of CaBFV and ICP (RPS). The relationships between elasticity and the ICP pulse shape–derived parameters were assessed using nonparametric Spearman rank correlation coefficient.

**Results:** Elasticity was positively correlated with ICP dominant pulse class: 0.43 (p = 0.018) and negatively correlated with P1/P2 and RPS: − 0.42 (p = 0.018) and − 0.55 (p = 0.002), respectively.

**Conclusion:** Our results show that ICP pulse class, P1/P2, and RPS at baseline are correlated with elasticity, suggesting that analysis of the shape of ICP pulse at baseline may reflect altered CSF hydrodynamics without the need for volumetric manipulation. Research supported by the Polish National Agency for Academic Exchange (International Academic Partnerships program).

### O02 Assessing the utility of newly proposed evans’ index cutoffs for use in normal pressure hydrocephalus

#### Alexander Davis^1^

##### ^1^Johns Hopkins University

###### **Correspondence:** Alexander Davis (adavi152@jhmi.edu)

*Fluids Barriers CNS* 2021, **18(2)**: O02

**Objective:** To assess the predictive value of the newly proposed Evans’ Index cutoffs in patients presenting with suspected Normal Pressure Hydrocephalus.

**Methods:** A retrospective chart review of 194 patients who underwent a cerebral spinal fluid tap test (CSF TT) at the Johns Hopkins Center for CSF Disorders was completed. Patients were divided into two groups: 130 patients were classified as having an EI above the ADNI cutoff (AC), and 64 patients were classified as having an EI below the ADNI cutoff (BC). Patients’ gait was assessed before and after the CSF TT using the 10 Meter Walk Test, Timed Up & Go, Dual Timed Up & Go, 6-Minute Walk Test, Mini-Balance Evaluation Systems Test. To assess the normality of the measures the Shapiro-Wilk test was used. The Wilcoxon rank-sum test and t-tests was used to assess between groups differences. The Wilcoxon matched-pairs signed-rank test and paired t-test were used assess within group differences. An ANCOVA controlled for age, sex, assistive device used, and past medical history effecting gait was used to assess the between groups response.

**Results:** There was not a significant difference in response between the patients above the new cutoffs and below the cutoffs. The patients below the cutoffs improved on the timed up and go (TUG) by 13.30%, DualTUG: 14.36%, 10-meter Walk Test (10MWT): 12.52%, MiniBEST: 18.94%, and 6-minute Walk Test (6MWT): 14.84%, while the patients above the new cutoffs improved on the TUG by 16.24%, DualTUG: 17.70%, 10MWT: 16.68%, MiniBEST: 17.85%, and 6MWT: 16.96%.

**Conclusion:** We recommend not adopting the newly proposed EI cutoffs for the differential diagnosis of NPH. The EI is primarily used as a screening tool to selecting patients for a CSF TT. Patients below the proposed cutoffs were shown to improve at the same levels as patients above below the cutoffs.

### O03 Association of pulsatile intracranial pressure with tonsillar descent and syringomyelia in chiari malformation I

#### Linda D’Antona^1^, Ruth Verity Passchier^1^, Claudia Craven^1^, Lucia Darie^1^, Lewis Thorne^1^, Laurence Watkins^1^, Ahmed Toma^1^

##### ^1^Victor Horsley department of Neurosurgery, National Hospital for Neurology and Neurosurgery, London, UK

###### **Correspondence:** Linda D’Antona (lindadantona@me.com)

*Fluids Barriers CNS* 2021, **18(2)**: O03

**Introduction:** The aim of this exploratory observational study was to describe the relationship between static/pulsatile intracranial pressure(ICP) and radiological findings in patients with CMI.

**Methods:** Single-centre retrospective observational study including a consecutive series of CMI patients investigated with elective 24-hour ICP monitoring. Night 24-hour ICP and pulse amplitude (PA) were retrieved from a prospectively built ICP monitoring database. Information on syringomyelia (present/absent), cerebellar tonsils descent(mm) and previous surgical treatments was collected. The association of ICP/PA and syringomyelia, tonsillar descent, previous shunt or previous foramen magnum decompression(FMD) were tested(Mann-Whitney U test or linear regression model).

**Results:** Thirty-six patients were included (33F, mean age was 36 ± 13 SD) years. Fourteen patients received previous treatment with foramen magnum decompression (n = 6), ventriculoperitoneal shunt (n = 1), or both (n = 7) before the time of ICP monitoring. Night ICP was not associated with any of the imaging findings investigated in this study. Night pulse amplitude had a significant association with tonsillar descent in patients without previous history of surgical treatment (β = 1.74, 95% CI 0.13 to 3.35, p = 0.03, adj. R2 = 0.16). In patients who did not improve despite previous ventriculoperitoneal shunt and foramen magnum decompression treatments (ultra-resistant CM I), there was a significant correlation of night pulse amplitude with syringomyelia length(in mm: β = 70, 95% CI 45 to 94, p = 0.001, and number of vertebral levels: β = 3.06, 95% CI 1.78 to 4.34, p = 0.002).

**Conclusion:** This exploratory study found an association between intracranial compliance and imaging findings of CMI. The findings of this exploratory study provide suggestions on the pathophysiology of CMI and are relevant for the planning of further research in this field.

### O04 Cerebrospinal fluid production rate in various pathological conditions: a preliminary study

#### Dr Kanza Tariq^1^, Mr Mohamed A. Elborady^1^, Linda D’Antona^1^, Lucia Daria^1^, Eleanor M. Moncur^1^, Mr Ahmed Toma^1^, Mr Lewis Thorne^1^, Mr Laurence Watkins^1^

##### ^1^National Hospital for Neurology and Neurosurgery, Queen Square, London, U.K

###### **Correspondence:** Kanza Tariq (kanzaharisqureshi@outlook.com)

*Fluids Barriers CNS* 2021, **18(2)**: O04

**Introduction:** Cerebrospinal fluid (CSF) production rate in humans is not clearly defined but is thought to be 18-24ml/hour. We recorded CSF production rate (PRcsf) in different pathological conditions using LiquoGuard, an automated CSF drainage machine. We analysed the data to see if CSF over-production is a feature of common neurosurgical conditions.

**Methods:** We performed a prospective observational study in all patients in our hospital who required CSF drainage through lumbar drain or external ventricular drain as part of their ongoing treatment. The external drain was connected to a LiquoGuard7 (Möller-Medical, Germany) with the intracranial pressure (ICP) sensor at the level of the external auditory meatus. Patients were flat for 10 minutes during the measurement. The study was repeated for 3 consecutive days. Statistical analysis used SPSS (version 25.0, IBM) by unpaired t-test, comparing measured rates to 20ml/hour.

**Results:** To date, we have calculated PRcsf in 37 patients. All patients suffering from a particular disease had similar results regardless of age, gender or co-morbidities.

**Conclusion:** PRcsf is higher than expected in many conditions which may have implications for decisions on CSF diversion. More extensive studies are needed to validate this technique.

**Table 1:** CSF production rate calculation of 37 patients

**Disease**	Normal Pressure Hydrocephalus	Post-surgical CSF leak	Sub-Arachnoid Haemorrhage	Intracerebral Haemorrhage	Spinal Lesions	Pituitary Adenomas
Number of Patients Studied	11	4	13	2	3	4
Average CSF production rate	55–69 ml/ hour	75–90 ml/ hour	150 ml/ hour	150 ml/ hour	100–150 ml/hour	26–36 ml/ hour
P value	(p < 0.0001)	(p < 0.0001)	(p < 0.0001)	(p < 0.0001)	(p = 0.0032)	(p = 0.049)

### O05 Change in pituitary size as an indicator of disordered csf dynamics in normal pressure hydrocephalus

#### Emanuele Camerucci^1^, Jonathan Graff-Radford^2^, David T. Jones^2^, Benjamin D. Elder^3,4^, Jeffrey L. Gunter^1^, Jeremy Cutsforth-Gregory^2^, Hugo Botha^2^, Matthew C. Murphy^1^, Clifford R. Jack Jr^1^, John Huston III^1^, Petrice M. Cogswell^1^

##### ^1^Department of Radiology, Mayo Clinic, Rochester, MN, 55905, USA;^2^Department of Neurology, Mayo Clinic, Rochester, MN 55905, USA; ^3^Department of Neurologic Surgery; ^4^Department of Physiology and Biomedical Engineering

###### **Correspondence:** Emanuele Camerucci (camerucci.emanuele@mayo.edu)

*Fluids Barriers CNS* 2021, **18(2)**: O05

**Introduction:** Idiopathic normal pressure hydrocephalus (iNPH) is a subtype of non- obstructive hydrocephalus with normal cerebrospinal fluid (CSF) pressure. Despite a normal mean pressure, we hypothesize that increased pulse pressure results in a decrease in pituitary gland size that may be reversed following shunt placement, similar to changes seen in intracranial hypertension. The purpose of this study is to assess if pituitary gland size increases following shunt placement.

**Methods:** Inclusion criteria were a clinical diagnosis of iNPH, ventriculoperitoneal shunt placement, and at least one MRI before and after shunt placement, performed from January 2015 through March 2021. The pituitary gland height was measured in the middle one-third of the gland on midline sagittal T1-weighted imaging using a computerized tool. A left-sided Wilcoxon signed rank test was performed to assess for increase in pituitary height between the pre-shunt MRI and first post-shunt MRI, and similarly between the first post shunt and last post- shunt MRI.

**Results:** Of 137 patients identified, 19 were excluded due to motion artifacts or inconsistencies in MRI techniques. Among the remaining 118, the pituitary gland size increased by 12.1% (median 0.40 mm [Q1: 0.10; Q3: 0.83], p < 0.001) between the pre-shunt and first post-shunt MRI and by 5.4% (0.25 mm [Q1: 0.00; Q3: 0.58], p < 0.001) between the first and the last post-shunt MRI.

**Conclusion:** The size of the pituitary gland increases after shunt placement for treatment of iNPH and increases even further in the ensuing months. Findings likely represent the effects of disordered CSF dynamics on the gland.

### O06 Choroid plexus tight junction disruption in an experimental model of intraventricular hemorrhage and post-hemorrhagic hydrocephalus

#### Ayodamola Otun^1^, Leandro Castaneyra-Ruiz^1^, James P. McAllister II^1^, Albert Isaacs^1^, Alexis Hartman^1^, Maria Garcia-Bonilla^1^, Sarah Zwick^1^, David Limbrick^1,2^

##### ^1^Department of Pediatric Neurosurgery, Washington University, St. Louis, MO, 63,110, USA; ^2^St. Louis Children’s Hospital, St. Louis, MO, 63,110, USA

###### **Correspondence:** Ayodamola Otun (aotun@wustl.edu)

*Fluids Barriers CNS* 2021, **18(2)**: O06

**Introduction:** 80% of Post-hemorrhagic hydrocephalus (PHH) cases are preceded by intraventricular hemorrhage (IVH). Junctional changes in the ventricular zone occur in IVH/PHH, but the effects on the choroid-plexus (ChP) are not well understood. Blood-CSF-Barrier (BCSFB) is essential for the maintenance of osmotic gradient across the ChP. We hypothesized that tight junction disruption, associated with increased osmolality, occurs in the ChP in IVH/PHH.

**Methods:** Blood-treated ferrets with and without ventriculomegaly were classified as PHH and IVH, respectively. N = 10 controls (intacts or shams), 10 IVH, and 9 PHH; and ventricular volume (VV) were calculated from MRI scans. Osmolality of intracisternal CSF was measured. Tight- junction immunostaining was performed.

**Results:** Mean VV in control, IVH, and PHH were 39.8 ± 9.4 mm^3^, 32.7 ± 14.6 mm^3^, and 80.4 ± 16.5 mm^3^ (p = 0.001), respectively. ZO-1 intensity decreased in IVH (p = 0.0006) and PHH (p = 0.0003), and %blood vessels (BV) expressing ZO-1 decreased in IVH (p = 0.0006) and PHH (p = 0.0003). %BV expressing claudin 5 decreased in IVH (P ≤ 0.0001) and PHH (P = 0.005). Osmolality increased in IVH (294.0 ± 4.2 mmol/kg, p = 0.007) and PHH (310.0 ± 4.8 mmol/kg, p ≤ 0.0001) compared to control (272.3 ± 3.0 mmol/kg) and was positively correlated with VV (p = 0.005, r = 0.72).

**Conclusion:** Compared to controls, ChP tight junctions decreased, and CSF osmolality increased in both IVH and PHH suggesting that cytopathology occurs without ventriculomegaly. However, compared to IVH, only CSF osmolality increased in PHH and was correlated with VV suggesting osmolality may be involved in ventriculomegaly. Altogether, findings suggest possible BCSFB disruption in IVH/PHH and role of osmolality changes in the progression of PHH from IVH.

### O07 Choroid plexus-on-a-chip: a microfluidic in vitro model to study the effect of inflammation on the blood-cerebrospinal fluid barrier

#### Hariharan Prashant^1^, Schwerk Christian^4^, Schroten Horst^4^, Blazer-Yost Bonnie^3^, Harris Carolyn^1,2^

##### ^1^Department of Biomedical Engineering, Wayne State University, Detroit, MI, United States; ^2^Department of Chemical Engineering and Materials Science, Wayne State University, Detroit, MI, United States; ^3^Department of Biology, Indiana University - Purdue University Indianapolis, IN, United States; ^4^Mannheim Medical Faculty, University of Heidelberg, Children’s Hospital, Mannheim, Germany,

###### **Correspondence:** Hariharan Prashant (fj1852@wayne.edu)

*Fluids Barriers CNS* 2021, **18(2)**: O07

**Introduction:** A monolayer of choroid plexus epithelial cells (CPECs) surrounding a core of fenestrated capillaries together constitute the blood-cerebrospinal fluid barrier (BCSFB). This tissue, located in the ventricles of the brain, is responsible for a majority of cerebrospinal fluid (CSF) production, regulation of molecules into the brain, and neuro-immune surveillance. Transwell plates and explant cultures have become important in vitro tools to study how these functions are altered in pathologic conditions like hydrocephalus. We built on these existing models and leveraged microfluidic techniques to develop a 2-channel barrier-on-a-chip device that recapitulates the dynamic environment of the BCSFB.

**Methods & results:** The choroid plexus-on-a-chip was designed with channel dimensions informed by computational fluid dynamics and fabricated using 3D printing and soft lithography techniques. Briefly, a porous polyethylene terephthalate membrane was sandwiched between two pieces of polydimethylsiloxane, forming the luminal and abluminal compartments. CPECs grown in the abluminal compartment establish a tightly connected, low permeability monolayer. Syringe pumps were used to provide the mechanical cue of hemodynamic shear stress from the luminal side and low ventricular wall shear from the abluminal side. In this way, changes in the monolayer’s integrity can be tracked by quantifying FITC-Dextran leakage across the compartments. Additionally, a four-electrode setup incorporated into the chip can be used to record trans epithelial electrical resistance as a measure of barrier function.

**Conclusion:** Future work involves inflicting chemical injury to the BCSFB and testing hypotheses surrounding inflammation mediated BCSFB dysfunction in hydrocephalus.

### O08 Collaboration with local physicians is essential to recruiting undiagnosed idiopathic normal pressure hydrocephalus

#### Hisayuki Murai^1^, Toshimasa Shin^2^, Fumitaka Shinozaki^1^, Shiroh Ikegami^1^, Atsushi Fujikawa^1^

##### ^1^Department of Neurosurgery, Saiseikai Narashino Hospital, Narashino, Chiba, 275-8580, Japan; ^2^Department of Neurosurgery, Higashi Funabashi Hospital, Funabashi, Chiba, 274-0065, Japan

###### **Correspondence:** Hisayuki Murai (murai@chiba-saiseikai.com)

*Fluids Barriers CNS* 2021, **18(2)**: O08

**Introduction:** With the aging of population, the number of patients with iNPH is increasing. However, the number of patients undergoing shunt surgery is small, even though the disease can be successfully treated. I moved to present hospital in 2017, increased the number of shunt surgeries and made our hospital local center for hydrocephalus. I would like to describe how we have recruited iNPH cases.

**Methods:** Without experience it is difficult to realize iNPH. First, I treated a typical case to let the staff know iNPH. The cooperation of the Radiology, Rehabilitation and Neurology Department is indispensable to make a team. Since iNPH patients rarely come to the neurosurgical out-patient clinic directly, we visited local clinic or hospital to present iNPH. The number of shunted cases and previous doctors were analyzed.

**Results:** The number of operations for hydrocephalus had been about 10 cases per year previously, but it has exceeded 40 cases per year recently. It is more than chronic subdural hematoma cases. The previous doctors of the last 100 shunted cases of iNPH were as follows.There were 31 cases inside the hospital (1: directly to neurosurgery, 23: neurology, 7: emergency department (6 falls, 1 cerebral infarction)), and 61 cases from local institutions (12: neurosurgery, 36: neurology, 7: psychiatry, 3: internal medicine, 1: brain dock, 1: orthopedics, 1: cardiovascular surgery), and 8 cases from remote hospitals (5: neurosurgery, 3: Neurology).

**Conclusion:** To recruit iNPH cases, making a hydrocephalus team in the hospital and the cooperation with local doctors are important.

### O09 Correlation of 24 hour ICP monitoring analysis and ICP slow waves

#### Eleanor Moncur, Linda D’Antona, Lewis Thorne, Laurence Watkins, Ahmed Toma

##### ^1^University College London Hospital

###### **Correspondence:** Eleanor Moncur (Eleanor.moncur@gmail.com)

*Fluids Barriers CNS* 2021, **18(2)**: O09

**Background:** Intracranial pressure (ICP) slow wave definition and clinical significance are debatable. Identification of waveform abnormalities by simple visual inspection is still a common clinical practice in assessing ICP disturbances. The significance of these waves in health and disease is not clearly understood. Median 24 hour ICP and pulsatility provide a simple and reproducible way of assessing ICP in routine clinical practice. Values of 24 hour ICP have been correlated with agreed clinical reference points during lumbar puncture and absence of spontaneous retinal venous pulsations. These methods do not take slow waves into account.

**Methods:** This is a retrospective single-centre study assessing the association between slow wave activity on ICP monitoring with median ICP and pulsatility calculated using ICM+ software (Cambridge Enterprise). Slow wave activity was compared in patients with normal versus abnormal median ICP, and normal versus abnormal median pulsatility. Separate comparisons were carried out for daytime and night-time data and calculations used unpaired t-tests.

**Results:** 31 patients were included. Slow wave activity was detected in the majority of patientscover a significant proportion of the ICP recording period, including those considered to have normal ICP and pulsatility, both during the day and night-time. Elevated slow wave activity was statistically significantly associated with abnormally high pulsatility overnight (P < 0.0001) and during the day (p = 0.0004). It was not statistically significantly associated with ICP in the day (p = 0.3676) or night (p = 0.0517).

**Conclusion:** The results of this study suggest that slow waves can be present in patients with no clear ICP abnormality. However, increased presence is associated with impaired compliance. Simple visual inspection of ICP trace for presence or absence of slow waves could mislead the diagnosis of patients with ICP disorders.

### O10 CSF diversion in selected chiari malformation i patients improves the crowding of the foramen magnum and reduces syrinx volume

#### Lucia Darie^1^, Mohamed Elborady^1^, Edward W Dyson^1^, Linda D'Antona^1^, Vejay Vakharia^1^, Janice Yiu^2^, Lewis W Thorne^1^, Ahmed K Toma^1^, Laurence D Watkins^1^

##### ^1^Department of Neurosurgery, National Hospital for Neurology and Neurosurgery, University Clinic London Hospitals, London, UK; ^2^Medical student at University College London Medical School, London, UK

###### **Correspondence:** Lucia Darie (darielucia@yahoo.com)

*Fluids Barriers CNS* 2021, **18(2)**: O10

**Introduction:** This study's objective was to elucidate whether patients with Chiari I malformation (CM I) treated primarily with a ventriculoperitoneal shunt (VPS) improved clinically and by 2D morphometric and 3D volumetric MRI measurements of the foramen magnum and syrinx cavity, respectively.

**Methods:** This is a retrospective case series study of CM I patients treated with VPS insertion following confirmed raised intracranial pressure (ICP) on 24h ICP monitoring. Clinical and radiological presentation, treatment and outcome were derived from the clinical records. The ICP monitoring results include median intracranial pressure (mICP) and median pulse amplitude (mPA). The surface area of the foramen magnum and syrinx volume pre- and post-intervention was calculated with a 3D segmentation software.

**Results:** A total of 15 patients with CM I who received a VPS were identified. Six had associated syringomyelia. The median ICP was 6.96 mmHg (− 0.5 to 17.76 mmHg). The median mPA was 5.82 mmHg (3.3 to 9.75 mmHg). The most common symptom was headaches. None of the patients were lost to follow-up. 11 patients reported symptomatic improvement following VPS. Two patients were excluded from the imaging segmentation due to loss of scans. The mean proportion of the foramen magnum occupied by the cerebellar tonsils decreased following VPS insertion from 41% (+/- 0.09) to 34% (+/- 0.61) (p = 0.0134, Wilcoxon-signed-rank). The mean syrinx volume decreased following VPS insertion from 3282.8 (+/-4250.34) mm3 to 669.47 (+/- 790.35) mm3 (p = 0.06, Wilcoxon-signed-rank).

**Conclusion:** In selected CM I patients with raised ICP, VPS represents a feasible option that can lead to clinical improvement.

**Keywords:** Chiari I, syringomyelia, ventriculo-peritoneal shunt, intracranial pressure monitoring, compliance, outcome, headaches, image segmentation, volumetry, cerebrospinal fluid dynamics

### O11 Developing a multidisciplinary normal pressure hydrocephalus service: a quality improvement initiative

#### Samuel MT Jeffery^1^, Rupert Noad^2^, Susie Wolstenholme^3^, Aishah Hannan^2^, Cathryn Harries^2^, Alice Marler^2^, Tina O’Farrell^1^, Samiul Muquit^1^

##### ^1^South West Neurosurgery Centre, Derriford Hospital, Plymouth, PL6 8DH, UK, ^2^Department of Neuropsychology, Derriford Hospital, Plymouth, PL6 8DH, UK, ^3^Department of Physiotherapy, Derriford Hospital, Plymouth, PL6 8DH, UK

###### **Correspondence:** Samuel Jeffery (samuel.jeffery1@nhs.net)

*Fluids Barriers CNS* 2021, **18(2)**: O11

**Introduction:** With an increasing elderly population we anticipate the need to develop an efficient and responsive Normal Pressure Hydrocephalus (NPH) service. We initiated a quality improvement project consolidating resources and expertise into a joint multidisciplinary team. This paper outlines the operational aspects of the first cycle of improvement.

**Methods:** A retrospective audit of all patients with suspected NPH assessed by the neurosurgical department 2014–2018 was undertaken, collecting demographic, clinical and operational data from case notes and electronic records. Using Lean Six Sigma principles a new patient pathway including a one-stop multidisciplinary clinic involving neurosurgery, neuropsychology and physiotherapy was designed and implemented, with subsequent re-audit of the service.

**Results:** In the historic cohort of 117 patients, 26 underwent lumbar drain test and 27 infusion test with 32 proceeding to shunt. A further 54 patients had a shunt without formal diagnostic testing. 41 patients were seen in the new multidisciplinary one-stop clinic, and following clinical and neuropsychological assessment NPH was discounted in 17. Trial of CSF drainage was offered to 24 patients with 13 proceeding to shunt. Up to 50 outpatient appointments per year were saved and median length of stay for a lumbar drain test was reduced from 6 to 3 days. Median length of stay for shunt insertion reduced from 3 to 2 days.

**Conclusion:** Introduction of a multidisciplinary pathway led to better utilisation of outpatient and inpatient resources for patients with suspected NPH. Further development of the service to optimise the patient pathway and assess clinical outcomes is planned.

### O12 Diagnostic test accuracy of extended lumbar drainage and receiver operating characteristic (roc) curve analysis of the optimal threshold for positivity

#### Adam Nunn^1^, Kelly McManus^1^, Rebecca Hodnett^1^, Richard Edwards^1^

##### ^1^Department of Neurosurgery, Southmead Hospital, Bristol, UK

###### **Correspondence:** Adam Nunn (adam.nunn@nbt.nhs.uk)

*Fluids Barriers CNS* 2021, **18(2)**: O12

**Introduction:** Extended lumbar drainage (ELD) is regarded by many as a reliable test for shunt-responsive idiopathic normal pressure hydrocephalus (iNPH). However, recent evidence has suggested its negative predictive value may be low.

**Methods:** An institutional database of 741 consecutive new patients presenting to our service with possible/suspected NPH was interrogated to determine the diagnostic accuracy of extended lumbar drainage. Patients were excluded if < 60 or a potential secondary cause of NPH was identified. Gait and cognitive performance data were collected at baseline, pre- and post- ELD and at 3 and 12 months post-shunt insertion. The decision to shunt was not solely based on objective assessment of ELD outcome immediately after drain removal, but also on patient/carer reported improvement during a two-week diary exercise.

**Results:** A clear relationship was found between improvement in Raftopoulos score at ELD and at 12 months post-shunt insertion (co-efficient 2.3, P < 0.01) and also between change in MMSE at ELD and at 12 months (co-efficient 0.5, P < 0.01). Using a composite of gait and cognitive improvement, ELD had a PPV of 81% and an NPV of 39%. ROC curve analysis suggested that ≥36% improvement in Raftopoulos score and ≥2 points on MMSE were the most discriminating thresholds for positivity.

**Conclusion:** ELD, if using objective measures alone, has a relatively high PPV but a low NPV, suggesting it is most useful in demonstrating the degree of improvement a patient may experience and aiding decision-making in patients who are undecided, rather than as a ‘rule out’ test for shunt insertion.

### O13 Enhanced hydrocephalus literature searches

#### Paul Relkin, Norman Relkin

##### ^1^Mapscallion LLC, New Jersey, USA

###### **Correspondence:** Norman Relkin (nrmdphd@gmail.com)

*Fluids Barriers CNS* 2021, **18(2)**: O13

**Introduction:** Searches of the peer-reviewed scientific literature are fundamental to evidence-based hydrocephalus research. The PubMed search engine is extremely powerful for this purpose, but comprehensive literature searches using PubMed’s platform are time- consuming and sometimes fail to identify relevant articles. Recent advances in machine learning may improve searches of the hydrocephalus literature.

**Methods:** We used machine learning techniques to embed the entire PubMed corpus of over 33 million articles in a high dimensional vector space. We searched within the embedding for “hydrocephalus” and created a lower dimensional representation that allows interactive exploration of the results using cluster analysis, geo-temporal mapping and filtering by subtopic search terms. To evaluate utility, we compared our search results with those listed in published meta-analyses and reviews. In addition, we used geo-temporal mapping to identify trends in the hydrocephalus literature over time and space.

**Results:** Through April 2021, a total of 35,320 published articles on the topic “hydrocephalus” were found. A surprising proportion of the retrieved literature related to secondary causes of hydrocephalus, including neoplasms, infections, vascular disease and metabolic causes. Emerging topics in the adult, pediatric and experimental hydrocephalus fields were readily identified. The number and relatedness of the retrieved articles compared favorably to those in several published hydrocephalus reviews.

**Conclusion:** Machine learning techniques show promise for improving the quantity and quality of hydrocephalus articles found in online literature searches. A demonstration version of our search tool that allows investigators to explore the results of the “hydrocephalus” search can be accessed at: bit.ly/hydro2021

### O15 EVD management, what’s right and what’s wrong

#### Joanna Palasz^1^, Anand Pandit^1,2^, Linda D’Antona^1,2^, Ahmed Toma^1^

##### ^1^Victor Horsley Department of Neurosurgery, National Hospital for Neurology and Neurosurgery, University College London Hospitals NHS Foundation Trust, London, UK; ^2^UCL Queen Square Institute of Neurology, London, UK

###### **Correspondence:** Joanna Palasz (jopalasz@gmail.com)

*Fluids Barriers CNS* 2021, **18(2)**: O15

**Introduction:** Little is known regarding the influence of the drain type and system on certain complications. Our aim is to evaluate the effects of different drain types, namely: bolt EVD vs. tunneled EVD vs. LD and system (Becker vs. LiquoGuard®), on complications associated with this procedure: CSF leakage, drain blockage and dislodgment.

**Methods:** We performed a prospective cohort study of EVDs or LDs inserted between January 2020 and March 2021.

**Results:** 106 drains were placed: 60 bolt, 24 tunneled, and 17 LDs. In 72 patients (mean age = 59 [SD 13], F = 24), 42 were placed in theatre, and 28 in SITU, all connected to the following CSF systems: Becker (39), LiquoGuard^®^ (14), or both (19). Diagnoses were: 49 SAH, 15 ICH, 6 tumours, and 2 other. Both drain type (p = 0.05, F = 3.05) and system (p = 0.003, F = 6.02) had a significant effect on blockage risk with tunneled-EVDs, having the highest rates of blockage (37%), as compared to bolt-EVDs (30%), and lumbar drains (5.9%). Once a drain had become blocked, there was a 58% chance for further blockage. Drain type was associated with duration of drainage (p = 0.004, F = 5.8), with LDs being removed faster than bolt (p = 0.002) or tunneled-EVDs (p = 0.009). Multinomial regression demonstrated a tendency for drain type to influence risk of CSF leak (p = 0.11, B = 0.78), with EVDs overall having less risk of leakage.

**Conclusion:** Drain type and system can influence the risk of drain-related complications. Further data collection and analysis is underway to elucidate the influence of these devices on CNS infection and vasospasm.

### O16 EVD-dependent hydrocephalus? An unconventional bargain with ICP

#### S. Krishnamurthy^1^, D. Gangoli^2^, A. Jagadeesh^3*^, S. Ganapathy^4^

##### ^1^Senior Resident, Department of Neurosurgery, St Johns Medical College, Bengaluru, India; ^2^Junior Resident, Department of Neurosurgery, St Johns Medical College, Bengaluru, India; ^3^Assistant Professor, Department of Neurosurgery, St Johns Medical College, Bengaluru, India; ^4^Assistant Professor, Department of Neurosurgery, St Johns Medical College, Bengaluru, India.

###### **Correspondence:** Surabhi Krisnamurthi (drsurabhikrishnamurthy@gmail.com)

*Fluids Barriers CNS* 2021, **18(2)**: O16

**Introduction:** According to Monro-Kellie doctrine, intracranial pressure (ICP) shares a unique relationship with the mean arterial pressure (MAP) and maintains the cerebral perfusion pressure (CPP) within normal limits. However, in this unique case, the patient needed subnormal ICP to maintain his GCS.

**Methods:** A 48-year-old male, known case of hemophilia A on factor VIII transfusions. He had undergone decompressive hemicraniectomy followed later by mesh cranioplasty in 2014 for spontaneous right frontotemporoparietal acute SDH with temporal hematoma. In November 2019, he underwent insertion of a medium pressure ventriculoperitoneal shunt for spontaneous right cerebellar hemorrhage with intraventricular extension with hydrocephalus. In January 2021, he presented with features of acute hydrocephalus with meningitis. Despite appropriate antimicrobial therapy and a functioning shunt system, his sensorium remained poor. EVD was inserted and ICP monitoring showed pressures within 12-15mmHg. It was noted that patient’s sensorium improved only at ICP levels of < 6mmHg.

**Results:** The patient was entirely EVD dependent even with revision of VP shunt chamber to very low-pressure chamber, requiring subnormal intracranial pressures to maintain sensorium. To tide over this tricky situation with EVD being only a temporary measure, a permanent solution in the form of gravity dependent CSF drainage was established by removing the VP shunt chamber thereby establishing a direct communication of the ventricles with peritoneal cavity. This helped in improving the patient’s sensorium while maintaining subnormal ICP levels.

**Conclusion:** This is a unique case where due to probable alteration in the cerebral compliance and autoregulation due to repeated insults to the brain, manifested as exponential reduction in CPP with minor changes in ICP though ICP and MAP were within physiological limits.

**Declarations**: The authors declare that the patient has give written consent for the publication of this study.

Figures and Charts:

**Fig. 1:**
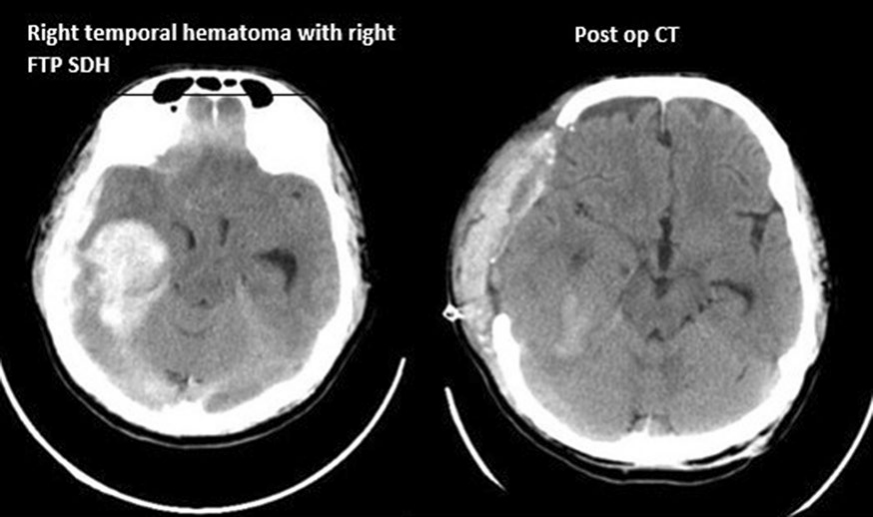
Right temporal hematoma with right FPP SDH and post op CT

**Fig. 2:**
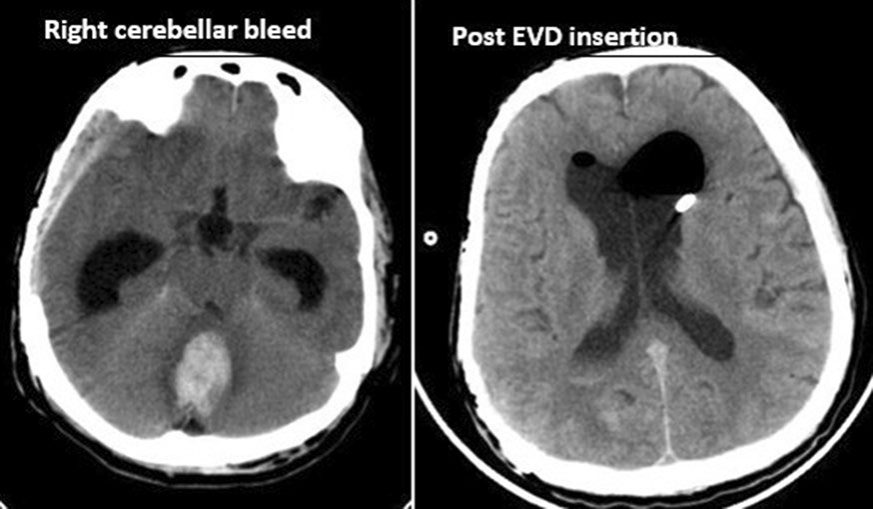
Right cerebral bleed and Post EVD insertion

**Fig. 3:**
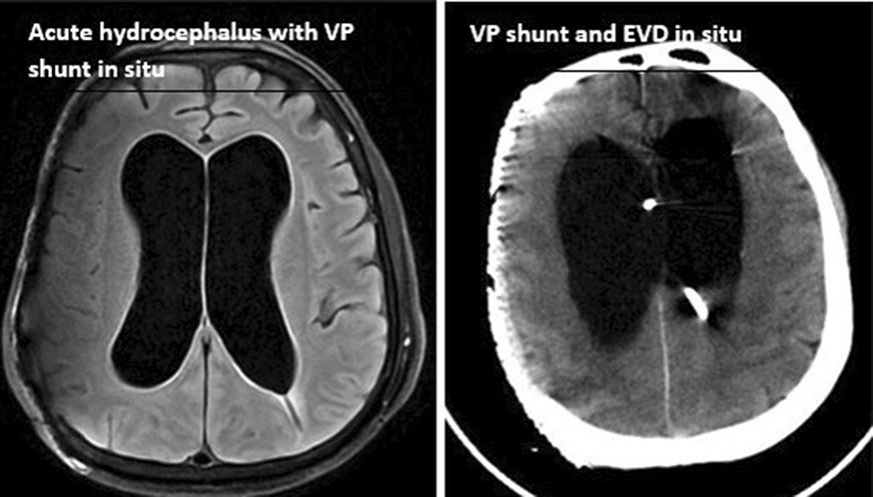
Acute hydrocephalus with VP shunt in situ, and VP shunt and EVD in situ

**Fig. 4:**
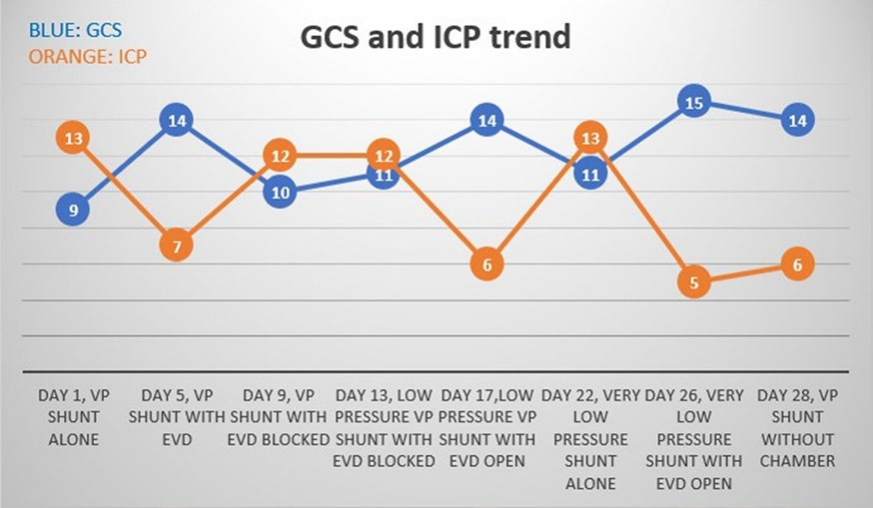
GCS and ICP trend

### O17 Experimental catheter lab; a rapid prototyping and in-vitro testing of novel ventricular catheters and catheter modifications

#### Ahmad Faryami^1^, Rooshan Arshad^2^, Dr. Carolyn A. Harris^3^

##### ^1^Biomedical Engineering, Wayne State University, Detroit, Michigan,48202 USA; ^2^School of Medicine, Wayne State University, Detroit, Michigan,48202 USA; ^3^Chemical Engineering and Material Science, Wayne State University, Detroit, Michigan,48202 USA

###### **Correspondence:** Ahmad Faryami (gw7895@wayne.edu)

*Fluids Barriers CNS* 2021, **18(2)**: O17

**Introduction:** Shunt-associated complications are extremely common. Computer- generated flow analysis reveals catheter limitations in terms of fluid dynamics, while in-vitro cell-attachment studies underline the importance of surface modifications to PDMS catheters. A high throughput rapid testing system is the combination of computer-generated flow analysis, experimental biomaterial analysis, protein adsorption and cell attachment and activation in vitro.

**Methods:** A pulsating flow system with custom-built control and data-collection software was developed. 2D flow-optimized chambers were printed using DLP technique with 1:1 model of various catheters built into the catheter. The models were made by curing a thin membrane of PDMS on the tangent of a set of catheter holes. Metal needles were placed through PDMS based on the model catheter design to avoid PDMS from curing over the holes. 3D models were also created by placing commercial catheters into the flow-optimized chambers. Novel catheters were created by accurately punching holes onto surface modified PDMS tubes and adding caps prior to placement into the chambers. 2D and 3D models were seeded with A1 and A2 astrocytes and were incubated with pulsating flow running through the chambers for up to 4 weeks prior to confocal microscopy.

**Results:** 2D and 3D models of novel ventricular catheters were connected to pulsating flow system. The flow system was able to induce controlled pulsation in the range of 40-230 bpm and 0.05-1 ml/min output.

**Conclusion:** This is a brief description of experimental catheter manufacturing and testing. 2D and 3D catheters allow rapid testing on novel catheter designs, surface modifications and biomaterials.

### O18 First 2-year experience from a pilot interdisciplinary normal pressure hydrocephalus clinic

#### Tobias Langheinrich^1,2^, Cliff Chen^3^, Owen Thomas^4^, Matthew Bailey^5^

##### ^1^Department of Neurology, Manchester Centre for Clinical Neurosciences, Salford Royal NHS Foundation Trust, M6 8HD, UK; ^2^Division of Neuroscience and Experimental Psychology, School of Biological Sciences, University of Manchester, M13 9PL, UK; ^3^Department of Neuropsychology, Manchester Centre for Clinical Neurosciences, Salford Royal NHS Foundation Trust, M6 8HD, UK; ^4^Department of Neuroradiology, Manchester Centre for Clinical Neurosciences, Salford Royal NHS Foundation Trust, M6 8HD, UK; ^5^Department of Neurosurgery, Manchester Centre for Clinical Neurosciences, Salford Royal NHS Foundation Trust, M6 8HD, UK

###### **Correspondence:** Tobias Langheinrich (tobias.langheinrich@srft.nhs.uk)

*Fluids Barriers CNS* 2021, **18(2)**: O18

**Introduction:** A departmental audit had recorded significant variations in care of patients suspected to have Normal Pressure Hydrocephalus (NPH). As a result an interdisciplinary neurological/neurosurgical clinic started in September 2018 at the Manchester Centre for Clinical Neurosciences (MCCN).

**Methods:** retrospective analysis of case notes of all patients referred to the NPH clinic. Referrer, number of patients who underwent or are awaiting tap test, number of patients who underwent or are awaiting shunting, as well as number of patients with DESH, LOVA, ventriculomegaly only and other (non NPH patterns) on MRI or CT brain were recorded. Note was made of the final clinical diagnosis and special populations such as those with co-morbidity potentially significantly contributing to their presenting symptoms to see whether patterns emerged.

**Results:** 237 patients were referred (20 by GP, 27 by physicians, 22 by psychiatrists, the remainder by neurologists or neurosurgeons). 84 underwent or are awaiting tap test, 41 have had or are awaiting VP shunt insertion, 69 had a DESH pattern, 48 had a LOVA pattern, 20 had no NPH pattern and the remainder had varying patterns of ventriculomegaly on MR brain.

**Conclusion:** The management of patients referred to the MCCN with suspected NPH is now standardised and diagnoses and treatment plans are formulated by an MDT. Patterns of patient populations such as those combining gait disorder and significant cognitive complaints, or gait disorder in the context of either confounding or contributing co-morbidity have emerged and can be managed efficiently with multidisciplinary expertise.

### O19 First insights of posture related pressure dynamics in awake and freely moving rats

#### Simone Schwander^1^, Fabian Flürenbrock^1,2^, Britta Bausch^3^, Anthony Podgoršak^1^, Petra Seebeck^4^, Melanie Zeilinger^2^, Marianne Schmid Daners^1^

##### ^1^Product Development Group Zurich, ETH Zurich, Zurich, 8092, Switzerland; ^2^Institute for Dynamic Systems and Control, ETH Zurich, Zurich, 8092, Switzerland; ^3^Interface Group, Institute of Physiology, University of Zurich, Zurich, 8057, Switzerland; ^4^Zurich Integrative Rodent Physiology, University of Zurich, Zurich, 8057, Switzerland

###### **Correspondence:** Simone Schwander (sischwan@ethz.ch)

*Fluids Barriers CNS* 2021, **18(2)**: O19

**Introduction:** To improve outcomes of current shunt treatments for hydrocephalus, a better understanding of cerebrospinal fluid (CSF) physiology is needed. Because malfunctions arise from posture changes, measurements of intracranial pressure (ICP) fluctuations and their relation to blood pressure during these changes shall provide valuable insights.

**Methods:** ICP and femoral blood pressure (FBP) of five healthy rats were continuously measured in a chronic trial via radio telemetry implants and sampled at 1 kHz. While being awake and moving freely in an observation box, the rats were monitored with a camera system at 30 fps. Means and correlation coefficients of ICP and FBP during ten natural rear ups per rat were analyzed with t-tests.

**Results:** Rear ups lasted on average 2.13 s. During these, FBP assessed as mean±SD (106.5 ± 17.4 mmHg) and ICP (1.4 ± 3.8 mmHg) were on average lower than FBP (118.9 ± 11.9 mmHg) and ICP (1.6 ± 4.0 mmHg) before rear ups. Changes of FBP were significant (p < 0.05) in all rats, whereas changes in ICP were significant (p < 0.05) in only two rats. In one of these two rats, correlation coefficients were significant (p < 0.01). ICP and FBP during these rear ups were on average moderately positively correlated (r = 0.24).

**Conclusion:** Concurrent measurements of CSF related pressures in rats are inherently challenging due to the limited space for sensor implants and rapid movements leading to strong artefacts. However, statistically significant CSF dynamics due to posture changes could be observed using high resolution pressure and video recordings.

### O20 Frequency of abdominal pain relative to various types of shunt tubing

#### Dr Kanza Tariq^1^, Mr Mohamed A. Elborady^1^, Dr Maria Kneizeh Al Geriass^1^, Linda D’Antona^1^, Lucia Darie^1^, Eleanor M. Moncur^1^, Mr Ahmed Toma^1^, Mr Lewis Thorne^1^, Mr Laurence Watkins^1^

##### ^1^National Hospital for Neurology and Neurosurgery, Queen Square, London, U.K

###### **Correspondence:** Kanza Tariq (kanzaharisqureshi@outlook.com)

*Fluids Barriers CNS* 2021, **18(2)**: O20

**Introduction:** Abdominal pain is a common complication of peritoneal shunt catheters and can lead to revision surgery in some patients. We aimed to analyse the frequency of intra- abdominal pain following peritoneal shunt insertion relative to the various types of shunt tubing according to manufacturer.

**Methods:** We performed a retrospective comparative study looking at the incidence of abdominal pain relative to peritoneal shunt catheters during the period of 2012 to 2020 in our hospital. Clinical data from 649 patients was evaluated. Only patient records with documented information about shunt tubing manufacturer were included. Activity level was evaluated through Modified Rankin Scale. Statistical analysis was done using SPSS (version 25.0, IBM) by Chi-Square test. A p-value < 0.05 was considered significant.

**Results:** After exclusion 426 patient records were examined. Ares abdominal catheter was found to be significantly associated with abdominal pain requiring revision surgery (p < 0.0001). Bactiseal shunt catheter had significantly low incidence of abdominal pain (p < 0.0001) in comparison to Ares. Modified Rankin Scale 0, 1 and 2 was associated with a higher incidence of abdominal pain.

**Conclusion:** Ares shunt tubing is associated with a higher incidence of abdominal pain and revision surgery following peritoneal shunt insertion

**Table 1:** Examination of 426 patient records to determine frequency of abdominal pain relative to various types of shunt tubing

Shunt Catheter According to Manufacturer	Number of patients studied	Average age	Number of Patients with abdominal pain	p value	Number of patients requiring revision surgery
Ares	174	65	78	p < 0.0001	10
Bactiseal	125	67	10	p = 0.314	1
Spiegelberg Silverline	40	69	6	p = 0.026	1
Plain tubing	87	45	13	p = 0.113	6

### O21 Happy-ICP: single-centre cohort of 184 telemetric intracranial pressure monitors

#### Ptolemy D W Banks, Anand S Pandit, Ahmed K Toma, Simon Thompson, Lewis W Thorne, Laurence D Watkins

##### ^1^Department of Neurosurgery, National Hospital for Neurology & Neurosurgery, London, UK

###### **Correspondence:** Ptolemy Banks (ptolemy.banks@nhs.net)

*Fluids Barriers CNS* 2021, **18(2)**: O21

**Introduction:** M-Scio^®^ (Christoph Miethke GmbH & Co. KG) is an implantable telemetric intracranial pressure (ICP) monitor used as part of a shunt system in the management of hydrocephalus patients. These tele-sensors could reduce the need for imaging surveillance and hospitalisation, and guide shunt valve adjustment. However, optimal ICP ranges remain unclear and the benefit of tele-sensors to patient care is uncertain.

**Methods:** Single-centre retrospective cohort study of patients with tele-sensors used as part of a cerebrospinal fluid (CSF) drainage system. Patient demographics, method of CSF diversion, ICP data, and pressure-related symptoms (headaches, nausea, visual abnormalities, and lethargy) were retrieved from the centre’s electronic health record. Patient presentation was categorised as either symptomatic or asymptomatic. ICP values were recorded in supine, sitting and standing positions. The frequency of hydrocephalus-related clinics/admissions was also recorded.

**Results:** 184 patients (69% female) had M-Scio tele-sensors implanted over 8 years (2012- 2021) for idiopathic intracranial hypertension (46%), secondary hydrocephalus (10%), or other indications (e.g., Chiari malformation). Procedures were predominantly a ventriculo-peritoneal (84%) or ventriculo-atrial (3.8%) shunt, or an endoscopic third ventriculostomy (3.8%). 31 patients had ICP measurements while asymptomatic averaging 10.2 mmHg (± 6.8) supine, − 8.3mmHg (± 4.9) sitting, and − 9.3 mmHg (± 5.6) standing. Over 4 years, tele-sensor patients averaged 1.9 (± 1.8) neurology and 4.7 (± 3.3) neurosurgery outpatient clinics, and 1.9 (± 1.9) neurosurgery and 0.4 (± 0.8) A&E admissions.

**Conclusion:** This data may help elucidate acceptable ICP ranges which can inform patient management. Tele-sensors may qualitatively and quantitatively improve service efficiency and patient experience.

### O22 Higher levels of neurofilament light chain and total tau in CSF are associated with negative outcome after shunt surgery in patients with normal pressure hydrocephalus

#### Madelene Braun^1^, Caroline Bjurnemark^2^, Dag Nyholm^1^, Valter Niemelä^1^, Kim Kultima^2^, Johan Virhammar^1^.

##### ^1^Department of Neuroscience, Neurology, Uppsala University Hospital, Sweden; ^2^Department of Medical Sciences, Clinical Chemistry, Uppsala University, Sweden

###### **Correspondence:** Madelene Braun (madelen.braun@akademiska.se)

*Fluids Barriers CNS* 2021, **18(2)**: O22

**Introduction:** Studies of negative predictors of shunt surgery outcome in patients with iNPH are scarce in the literature. The aim of this study was to investigate if CSF biomarkers can predict outcome after shunt surgery and if there were any associations between CSF biomarkers and symptoms.

**Methods:** Preoperative CSF biomarkers were analyzed in 455 patients with iNPH at a single center during 2011–2018. Symptoms were graded with the Swedish iNPH-scale before and 12 months after shunt surgery. Neurofilament light chain protein (NfL), total tau (T-tau), phosphorylated tau (P-tau) and amyloid beta1-42 (Aß1-42) were investigated. Preoperative evaluation and follow-up were available in 376 patients.

**Results:** A linear regression model established that higher levels of NfL and T-tau were associated with less improvement after shunt surgery, p < 0.05. Patients whose symptoms deteriorated after shunt surgery had higher preoperative levels of NfL, (p < 0.01) and T-tau (p < 0.05), and lower levels of Aß1-42 (p < 0.01). Of the patients who improved ≥ 5 levels in the iNPH-scale (55%), NfL was higher than reference range in 22%, T-tau in 8%, P-tau in 9% and Aß1- 42 was below reference range in 61% of the patients.

**Conclusion:** Higher levels of T-tau and NFL predict a less favorable response to shunt surgery. CSF levels can be elevated also in patients who respond to shunt surgery, consequently none of these CSF biomarkers can be used to exclude patients from surgery.

### O24 Impact of the COVID-19 pandemic in inph surgical practice. a care giver-based questionnaire survey

#### Tuniz Francesco^1^, Moreale Renzo^3^, Palazzese Paola^1^, Piccolo Daniele^1^, Fabbro Sara^1^, Belgrado Enrico^2^, Skrap Miran^1^

##### ^1^Neurosurgical Department, Santa Maria della Misericordia Hospital, Udine, 33100, Italy; ^2^Neurological Department, Santa Maria della Misericordia Hospital, Udine, 33100, Italy; ^3^Nursing Bachelor Degree Course, University of Udine, Udine, 33100, Italy

###### **Correspondence:** Francesco Tuniz (tuniz.francesco@gmail.com)

*Fluids Barriers CNS* 2021, **18(2)**: O24

**Introduction:** iNPH is a disease that leads to the progressive disability. Shunt procedures has been defined as the gold standard procedures allowing recovery. During the pandemic, our hospital has been transformed and all non-emergency surgical procedures have been postponed or cancelled. Retrospectively reviewing the amount of the neurosurgical activity performed at our clinic in the last year, we reported more than 50% decrease of surgeries due to the pandemic. The aim of this study was to analyze the impact of waiting on people with iNPH.

**Methods:** 30 caregivers were interviewed through an interview and a questionnaire consisting in 15 items investigating the patient’s cognition, walking and urinary domains. The score ranged from a minimum of 0 (dependent) to a maximum of 45 (independent).

**Results:** The mean questionnaire score was 31 (16–41) at the time of diagnosis and 18 (3–37) at the time of the interview (still in waiting-list). Patients lost on average 13.75 points (0–33) during the waiting time. 75% of patients required new aids, especially for mobilization. During the waiting period they made an average of 3.3 (0–11) visits to the hospital due to the worsening conditions.

**Conclusion:** Patients and caregivers can provide useful elements to reflect on the criteria we use to define the emergency of surgery. In situations where waiting times are prolonged, it is necessary to inform/help caregivers how to access services available at the community levels. Postponing surgery in iNPH patients could provide consequences for the QOL of patients and caregivers.

### O25 Improvement in the long-term care burden after surgical treatment of patients with idiopathic normal pressure hydrocephalus: a supplementary study

#### Masatsune Ishikawa^1,2*^, Shigeki Yamada^3,2^, Masakazu Miyajima^4^, Hiroaki Kazui^5^, Etsuro Mori^6^

##### ^1^Rakuwa Villa Ilios, Kyoto, Kyoto, Japan; ^2^Normal pressure hydrocephalus Centre, Otowa Hospital, Kyoto, Kyoto, Japan; ^3^Department of Neurosurgery, Shiga University of Medical Science, Otsu, Shiga, Japan; ^4^Department of Neurosurgery, Juntendo Tokyo Koto Geriatric Medical Center, Kotoku, Tokyo, Japan; ^5^Department of Neuropsychiatry, Kochi Medical School, Kochi University, Nankoku, Kochi, Japan ^6^Department of Behavioral Neurology and Neuropsychiatry, Osaka UniversityUnited Graduate, Toyonaka, Osaka, Japan

###### **Correspondence:** Masatsune Ishikawa (rakuwadr1001@rakuwadr.com)

*Fluids Barriers CNS* 2021, **18(2)**: O25

**Introduction:** Idiopathic normal pressure hydrocephalus (iNPH) is a surgically treatable syndrome commonly observed in older adults. However, it is unclear whether clinical improvements after surgery can effectively reduce the long-term care burden (LTCB). In this study, we determined whether shunt surgery was effective in decreasing LTCB. We also investigated the degree of variability in patients and hospitals, using data from the iNPH multicenter study.

**Methods:** This study involved 69 participants who underwent lumboperitoneal shunt surgery with follow-up for 12 months. A generalized linear mixed model was applied to analyze the fixed and random effects simultaneously.

**Results:** Regarding LTCB, the disability grades improved significantly. Although the dementia grades also improved, it was not statistically significant. The differences in the LTCB grades in most patients were within the range of the 95% confidence intervals, while in the case of hospitals, some were often out of the range.

**Conclusion:** Further studies are needed to improve dementia in patients with iNPH. The incorporation of random variables, such as hospitals, is important in for the analysis of data from multicenter studies.

### O26 Improvements of physical activity, functional strength and endurance and increased sleep at night after shunt surgery in idiopathic normal pressure hydrocephalus

#### Johanna Rydja^1^, Lena Kollén^2^, Per Hellström^2^, Carsten Wikkelsø^2^, Martin Ulander^3^, Mats Tullberg^2^, Fredrik Lundin^4^

##### ^1^Department of Activity and Health, and Department of Biomedical and Clinical Sciences, Linköping University, Sweden; ^2^Institute of Neuroscience and Physiology, Sahlgrenska Academy, University of Gothenburg, Sweden; ^3^Department of Clinical Neurophysiology, and Department of Biomedical and Clinical Sciences, Linköping University, Sweden; ^4^Department of Neurology, and Department of Biomedical and Clinical Sciences, Linköping University, Sweden.

###### **Correspondence:** Johanna Rydia (johanna.rydja@regionostergotland.se)

*Fluids Barriers CNS* 2021, **18(2)**: O26

**Introduction:** Despite documented improvements of gait in iNPH-patients following shunt surgery, no effect on the magnitude of spontaneous daily physical activity has been proven. As a part of our previously reported rehabilitation study (iNPHys) this study aimed to evaluate the effects of a 12-weeks postoperative exercise program on physical activity, functional strength, endurance and sleep.

**Methods:** Seventy-nine iNPH patients who had been randomized to either an exercise group (EG, n = 34) or a control group (CG, n = 45) with valid actigraphy recordings of physical activity and sleep were included in the study. Recordings were made during seven days preoperatively and three months and six months postoperatively. Endurance was evaluated with the 6-minute walk test and functional strength with the 30-second chair stand test.

**Results:** Between group differences, all in favour of the EG, were seen in changes from baseline to three months regarding improvement in functional strength (p = 0.011) and endurance (p = 0.033) and a slight reduction in daytime sleep (p = 0.033). None of these differences remained after six months. The whole group (i.e., EG and CG together) improved in steps per day (p = 0.023), functional strength (p < 0.001) and endurance (p<0.001) and the proportion of sleep at night was increased (p = 0.024) after three months. These changes remained after six months.

**Conclusion:** Shunt treatment of iNPH-patients improved spontaneous physical activity, functional strength and endurance. The proportion of sleep at night was increased. The effects remained after six months. Postsurgical participation in an exercise program had an additional short-term effect on functional strength and endurance.

### O27 In vitro investigation of the influence of cervical spinal canal stenosis on csf hydrodynamics

#### Anne Benninghaus^1^, Leonie Stellmann^1^, Uwe Kehler^2^, Klaus Radermacher^1^

##### ^1^Chair of Medical Engineering, RWTH Aachen University, Germany; ^2^Department of Neurosurgery, Asklepios Klinik in Altona, Hamburg, Germany

###### **Correspondence:** Anne Benninghaus (benninghaus@hia.rwth-aachen.de)

*Fluids Barriers CNS* 2021, **18(2)**: O27

**Introduction:** The impact of spinal stenosis on cerebrospinal fluid (CSF) dynamics is still unclear but can be observed in NPH patients. In particular, the correlation with the disease normal pressure hydrocephalus (NPH), respectively with its pathogenesis is vague. Therefore, the aim of this study was to experimentally investigate the influence of varying degrees of stenosis in the cervical region on CSF hydrodynamics with respect to NPH.

**Methods:** An in vitro model of the craniospinal CSF dynamics, developed in our lab, was used. The stenoses were located in the C6 region. The hydrodynamic cross-sectional area varied in seven measurements from 19.63 mm^2^ (no stenosis) to 0 mm^2^ (total blockage) to simulate different degrees of stenosis. Intracranial pressure (ICP), spinal flow and cranial and spinal compliance were measured.

**Results:** The results show an increase of the ICP amplitude from 4.94 mmHg (physiological/ no stenosis) to 7.51 mmHg (total blockage), which is a rise of 52.02 % and furthermore an accompanying decrease in overall compliance of 56.15%.

**Conclusion:** Increased ICP amplitudes and a decreased craniospinal compliance are typical characteristics of NPH patients. Nevertheless, it is not clear whether a spinal stenosis influences or favors the development of NPH. Therefore, clinical investigation should be performed to determine the prevalence and severity of spinal canal stenoses in NPH patients.

### O28 Incidence of idiopathic normal pressure hydrocephalus among patients with balance and gait disorders in memory clinic population

#### George Razay^1^

##### ^1^Dementia Research Centre, Department of Medicine, Launceston General Hospital, University of Tasmania, Launceston, Tasmania.

###### **Correspondence:** George Razay (george.razay@ths.tas.gov.au)

*Fluids Barriers CNS* 2021, **18(2)**: O28

**Introduction:** Balance and gait disorders (B&GDs) increase with ageing but often not adequately evaluated and largely underdiagnosed. We have therefore investigated the prevalence of B&GDs and the underlying diagnosis among patients with memory impairment.

**Methods:** 410 consecutive patients enrolled between 2010 and 2014 from the Memory Disorders Clinic, Launceston, Tasmania. All patients had detailed history of memory, balance and gait symptoms including features suggesting dementia. A full examination included Mini- Mental State Examination (MMSE) and balance/gait functions by standing with eyes closed, on toes, and the tandem test. All patients had brain CT scan.

**Results:** 218 women and 192 men participated in the study, median age 76.5 years (range 32.6-94.8) and mean MMSE score 23.3 (SD 4.9). 214 (53%) of patients had B&GDs. 214 (53%) had B&GIs of whom, 85 (40%) had mild cognitive impairment (MCI), 25 (12%) had Alzheimer’s disease (AD), 62(29%) had INPH, 11 (5%) had mixed dementia (MD), 10 (5%) had vascular dementia (VD), 9 (4%) had Parkinson’s disease dementia, 2(1%) had Lewy body disease (LBD). 196(48%) didn’t have B&GIs, of whom, 97 (50%) had MCI, 66 (34%) had AD, 13(7%) had MD, 6(3%) had Gertsmann’s syndrome, 4 (2%) had hypogonadism, and 4 (2%) had frontal lobe dementia (FLD). After excluding patients with MCI, patients with B&GIs (n = 129); 60 (48%) had INPH, 25 (20%) had AD, 11 (9%) had MD, 10 (8%) had VD, 2 (2%) had LBD. Patients without B&GDs (n = 100), 66% had AD, 13% had MD, 1% had VD and 4% had FLD.

**Conclusion:** The study shows that B&GDs are common among patients with memory impairment, and highlights the high prevalence of INPH, a condition that could be treated with shunt surgery with improvement of cognitive, balance and gait functioning.

### O29 iNPH and parkinsonism: a positive shunt response with a negative tap test

#### Paolo Mantovani^1^, Giulia Giannini^2^, David Milletti^3^, Sabina Cevoli^4^, Nicola Valsecchi^5^, Pietro Cortelli^2^, Giorgio Palandri^1^

##### ^1^U.O. Neurochirurgia, IRCCS Istituto delle Scienze Neurologiche di Bologna, Bologna, 40139, Italy; ^2^Dipartimento di Scienze Biomediche e Neuromotorie DIBINEM, Università di Bologna, Bologna, 40126, Italy; ^3^U.O.S.I. Medicina Riabilitativa, IRCCS Istituto delle Scienze Neurologiche di Bologna, Bologna, 40139, Italy; ^4^Clinica Neurologica Metropolitana NEUROMET, IRCCS Istituto delle Scienze Neurologiche di Bologna, Bologna, 40139, Italy; ^5^Dipartimento di Medicina Specialistica Diagnostica e Sperimentale DIMES, Università di Bologna, Bologna, 40139, Italy

###### **Correspondence:** Paolo Mantovani (paolmantovani@gmail.com)

*Fluids Barriers CNS* 2021, **18(2)**: O29

**Introduction:** Parkinsonism in Idiopathic Normal Pressure Hydrocephalus is reported with a highly variable prevalence (20-86%), so it should be carefully evaluated as it may represent an additional symptom, a feature of a neurodegenerative disease mimicking the clinical picture of INPH, or the manifestation of a second co-existing pathology. Tap Test is a useful tool for the diagnosis of INPH and gives important information for shunt surgery indication. In this study, selected probable INPH patients, with and without parkinsonism were compared at baseline evaluation, 72 hours after tap test and months after surgery.

**Methods:** 64 patients with probable INPH who underwent shunt surgery were selected by the Prohydro team in Bologna. Among these, 12 patients (18,75%) fulfilled diagnostic criteria for parkinsonism. Considered clinical variables were Timed Up & Go time, Tinetti scale for gait, balance and fall risk, INPH Grading Scale and Modified Rankin Scale for overall disability.

**Results:** At baseline, patients with parkinsonism (INPH-P) had worse clinical scores and disability than patients with no parkinsonism (INPH-NP). INPH-NP patients showed a significant clinical improvement after tap test and shunt surgery in most considered variables. INPH-P patients, on the other hand, did not have any significant change in any considered variable after tap test, while a significant improvement was recorded six months after surgery.

**Conclusion:** The particular profile of INPH patients with parkinsonism should be carefully considered when eligibility for shunt surgery is discussed, as in our series a negative tap test is not associated with a negative response to shunt surgery.

### O30 Intercompartmental communication in the cerebrospinal fluid system in an acute ovine *in-*vivo trial: first impressions

#### Anthony Podgoršak^1^, Nina Trimmel^2^, Markus Oertel^3^, Margaret Arras^2^, Sara Qvarlander^4^, Anders Eklund^4^, Miriam Weisskopf^2^, Marianne Schmid Daners^1^

##### ^1^Department of Mechanical and Process Engineering, ETH Zurich, Zurich, Switzerland; ^2^Center for Surgical Research, University Hospital Zurich, University of Zurich, Zurich, Switzerland; ^3^Department of Neurosurgery, University of Zurich, Zurich, Switzerland; ^4^Department of Radiation Sciences, Umea University, Umea, Sweden.

###### **Correspondence:** Anthony Podgorsak (apodgorsak@ethz.ch)

*Fluids Barriers CNS* 2021, **18(2)**: O30

**Introduction:** While there have been decades of research dedicated to the mechanisms behind cerebrospinal fluid (CSF) dynamics, there are still knowledge gaps. The important pressure communications between spinal and intracranial compartments, including lag times, are sparsely studied and are invaluable to create more complete models.

**Methods:** An *in-vivo* trial in sheep (n = 6) was conducted to quantify the intercompartmental communication existing within the CSF system. Standardized infusion testing was performed, including bolus and constant pressure infusions (CPI). Bolus infusions contained six lumbar infusions of 0.5 mL Ringer’s solution. CPI were comprised of six regulated pressure steps of 3.75 mmHg for periods of 7 min each. Intracranial reaction lag times to infusions were calculated via cross-correlation, pressure changes and the respective Rout calculated (1 kHz sampling frequency).

**Results:** The study was successfully conducted on a novel CSF animal model. Four of six sheep reacted to the intrathecal pressure increase. The respective increase propagated across the CSF system cranially for the bolus infusion with a mean intracranial pressure change, lag, and Rout of 15.3 ± 1.3 mmHg, 131 ± 7 ms, and 46.5 ± 4.2 mmHg*mL^-1^min, respectively, and for the CPI of 15.4 ± 1.5 mmHg, 122 ± 19 ms, and 77.8 ± 3.0 mmHg*mL^-1^min respectively.

**Conclusion:** Standardized infusion tests with multi-compartmental pressure recordings in sheep have helped capture distinct reactions between the intrathecal and intracranial compartments of the CSF system. Interestingly we found no communication in two of six sheep, which needs to be further investigated. These results represent an important first step into improvements in current CSF modelling methodology.

### O31 Intra-abdominal fixation of lumboperitoneal shunt to prevent proximal migration in cases of idiopathic intracranial hypertension: technical note

#### A.R. Rizk^1^, C. Becker^2^, M. Bettag^1^

##### ^1^Department of neurosurgery, Krankenhaus der Barmherzigen Brüder Trier, Trier, Germany; ^2^Department of Surgery, Krankenhaus der Barmherzigen Brüder Trier, Trier, Germany

###### **Correspondence:** Ahmed Rizk (arizkrizk@gmail.com)

*Fluids Barriers CNS* 2021, **18(2)**: O31

**Introduction:** We performed laparoscopicC assisted lumboperitoneal (LP) shunt placement through a lateral abdominal approach for cases of idiopathic intracranial hypertension (IIH). Shunt migration is one of the most frequent complications of LP shunts. Especially in the lateral abdominal approach, migration of the distal end of the catheter outside the peritoneal cavity is more frequent because of the difficulty of fixing the catheter to the abdominal wall in obese patients treated in the lateral position.

**Methods:** We present here a special technique in which we used hemostatic clips to fix the distal end of the peritoneal catheter to the uterosacral ligament in two patients treated after migration of the catheter outside the peritoneal cavity into the subcutaneous space.

**Technique & results:** The surgery is performed in the right lateral decubitus position. After introduction of the lumbar end of the shunt into the subarachnoid space, the catheter is anchored to the lumbodorsal fascia to help prevent migration. Simultaneously, access to the peritoneal cavity is performed by the laparoscopic surgeon. Then a split trocar is placed under laparoscopic visualization through a separate stab incision in the left flank. The catheter is tunneled subcutaneously from the lumbar area to the left flank and then under direct laparoscopic visualization the catheter is introduced into the peritoneal cavity through the split trocar. The peritoneal catheter is anchored to the uterosacral ligament using 2 hemostatic clips at the most distal end of the catheter. The patency of the shunt is checked by observing the free flow of CSF through its distal end.

**Keywords:** Lumboperitoneal shunt C Proximal migration C LaparoscopicC assisted C idiopathic intracranial hypertension

### O32 Intracranial cerebro-spinal fluid (CSF) diversion from 4^th^ ventricle to cisterna magna

#### Pradnya Patkar^1^, Sushil Patkar^2^

##### ^1^Department of Neurosciences, Royal Preston Hospital, PR2 9HT, UK; ^2^Department of Neurosurgery, Poona Hospital & Research Centre, Pune 411030, Maharashtra, India

###### **Correspondence:** Pradnya Patkar (pradnyapatkar04@gmail.com)

*Fluids Barriers CNS* 2021, **18(2)**: O32

**Introduction:** Establishing site of obstruction and patency of bulk flow CSF pathways is important in evaluation of obstructive hydrocephalus. Re-establishing intracranial CSF flow is superior to extracranial CSF diversion.

**Methods:** *Case 1*: A 13-year-old girl presented with 15 days history of mild headaches, occasional vomiting, diplopia, and ataxia, 7 years after successful ventriculo-peritoneal shunt (VPS) insertion for post-meningitic hydrocephalus. Magnetic resonance (MR) imaging and CT ventriculography with an iohexol (a water-soluble, non-ionic contrast) revealed a disproportionately enlarged, entrapped 4th ventricle. Patent basal cisterns were confirmed with basal cisternography. *Case 2*: A 72-year-old male presented with a 2-day history of altered sensorium and refusal to feed following a mechanical fall 7 years after successful VPS insertion for post-meningitic hydrocephalus. MR imaging revealed acute pan-ventricular hydrocephalus with ballooning of bilateral foramen of Luschka. Shunt series imaging revealed retro-auricular disconnection of the shunt tubing with complete migration into the pelvic peritoneal cavity. Basal cisternography confirmed patency of bulk flow CSF pathways. Both patients underwent diversion of CSF from 4^th^ ventricle into cisterna magna with a silastic catheter.

**Results:** Both patients had instantaneous symptomatic relief and follow up imaging at 3 weeks revealed resolution of radiological abnormalities.

**Conclusion:** Intracranial CSF diversion is superior as both ends of the catheter are subjected to the same pressure dynamics. Over-drainage, disconnection and infection are established complications of extracranial CSF diversion which can be avoided by this method. This is a safe, simple, low-cost and reasonably applicable technique which does not require any special equipment.

**Declarations**: The authors declare that the patient has give written consent for the publication of this study.

### O33 Intracranial pressure dynamics when moving from sitting to standing

#### Matthew J. Bancroft^1,2^, Eleanor Moncur^3^, Amy L. Peters^2^, Linda D’Antona^3^, Lewis W. Thorne^3^, Laurence D. Watkins^3^, Brian L. Day^2^, Ahmed K. Toma^1,3^

##### ^1^Department of Brain Repair and Rehabilitation, UCL Queen Square Institute of Neurology, London, WC1N 3BG, United Kingdom; ^2^Centre for Behavioural and Vestibular Neurosciences, Department of Clinical and Movement Neurosciences, UCL Queen Square Institute of Neurology, London, WC1N 3BG, United Kingdom; ^3^Victor Horsley Department of Neurosurgery, National Hospital for Neurology and Neurosurgery, Queen Square, University College London Hospitals, London, WC1N 3BG, United Kingdom

###### **Correspondence:** Matthew Bancroft (matthew.bancroft.13@ucl.ac.uk)

*Fluids Barriers CNS* 2021, **18(2)**: O33

**Introduction:** Humans frequently change posture in daily life but the relationship between posture and intracranial pressure (ICP) is poorly understood. ICP dynamics when transitioning from a lying to sitting posture are relatively well studied but other movements are less so. Here we present findings on ICP dynamics when moving from a sitting to standing posture.

**Methods:** We recruited ambulatory patients either with (n = 4) or without a shunt (n = 7) who were undergoing ICP monitoring as part of their clinical work-up at the National Hospital for Neurology and Neurosurgery, London. Patients sat upright on a chair with their head in a neutral position and moved to and from a standing posture at 20 second intervals. ICP was recorded at 100Hz using a parenchymal ICP monitor (Neurovent P Raumedics). Postural data was acquired at 100Hz by inertial measurement units (MTw Awinda, Xsens, Netherlands) attached to the chest and thigh. We compared mean ICP when sitting and standing.

**Results:** ICP increased significantly when moving from a sitting to standing posture (F(1,9) = 10.6; p = 0.01; β = 0.83; 95% confidence interval of mean difference: 0.6–3.5 mmHg). No differences in ICP dynamics were detected between shunted and non-shunted patients (group: F(1,9) = 0.3, p = 0.58; β = 0.06; group x posture: F(1,9) = 0.1, p = 0.74; β = 0.06), although at the time of submission the study is underpowered to detect any differences.

**Conclusion:** Transitioning from a sitting to standing posture induces an increase in ICP. Future work should determine the effect of shunting on ICP dynamics during sit-to-stand transitions.

### O34 Is hydrocephalus shunting functional neurosurgery?

#### Eric Schmidt^1^, Fabien Despas^2^, Anne Pavy le Traon^3^, Marek Czosnyka^4^, John D Pickard^4^, Kamal Rahmouni^5^, Atul Pathak^2^, Jean Michel Senard^2^

##### ^1^Department of neurosurgery, Toulouse, France; ^2^Department of Clinical Pharmacology, Toulouse, France; ^3^Department of Neurology, Toulouse, France; ^4^Department of Neurosurgery, Cambridge, UK; ^5^Departments of Pharmacology, University of Iowa, Iowa City, Iowa, USA

###### **Correspondence:** Eric Schmidt (schmidt.e@chu-toulouse.fr)

*Fluids Barriers CNS* 2021, **18(2)**: O34

**Introduction:** Functional neurosurgery aims at modulating the function of neural networks involved in movement, spasticity, pain or behavior. Hydrocephalus shunting aims at restoring neurological function by improving brain fluid mechanics and intracranial pressure (ICP). We hypothesize that hydrocephalus shunting is functional neurosurgery. To validate this hypothesis, one must demonstrate the presence of i) an intracranial barosensitivity, ii) intracranial baroreceptors and integrating centers and iii) a shunt-induced neuromodulation with a *pari passu* clinical response. This work is designed to explore the first step.

**Methods:** Modest ICP increase and decrease were achieved in mice and patients with intra-ventricular and lumbar fluid infusion. Sympathetic activity was gauged directly by microneurography, recording renal sympathetic nerve activity in mice and muscle sympathetic nerve activity in patients. Heart-rate variability analysis was also performed in both species.

**Results:** In mice (n = 15), renal sympathetic activity increased from 29.9 ± 4.0 bursts.sec^-1^ (baseline ICP 6.6 ± 0.7 mmHg) to 45.7±6.4 bursts.sec^-1^ (plateau ICP 38.6 ± 1.0 mmHg) and decreased to 34.8 ± 5.6 bursts.sec^-1^ (post-infusion ICP 9.1 ± 0.8 mmHg). In patients (n = 10), muscle sympathetic activity increased from 51.2 ± 2.5 bursts.min^-1^ (baseline ICP 8.3 ± 1.0 mmHg) to 66.7 ± 2.9 bursts.min^-1^ (plateau ICP 25 ± 0.3 mmHg) and decreased to 58.8 ± 2.6 bursts.min^-1^ (post- infusion ICP 14.8±0.9 mmHg). Heart-rate variability analysis demonstrated a significant vagal withdrawal during the ICP rise, in accordance with the microneurography findings. Mice and human results are alike.

**Conclusion:** We demonstrate in animal and human that ICP is a reversible determinant of neuronal efferent sympathetic outflow, even at relatively low ICP levels. Our work supports the presence of an intracranial barosensitivity.

### O35 Is there an expiry date for shunts in NPH?

#### Aida Kafai Golahmadi^1,2^, Eleanor Moncur^2^, Linda D’Antona^2^, Lewis Thorne^2^, Laurence Watkins^2^, Ahmed Toma^2^

##### ^1^School of Medicine, Imperial College London, London, SW7 2BX, UK; ^2^Department of Neurosurgery, The National Hospital for Neurology and Neurosurgery, London, WC1N 3BG, UK

###### **Correspondence:** Aida Kafai Golahmadi (aida.kafai-golahmadi17@imperial.ac.uk)

*Fluids Barriers CNS* 2021, **18(2)**: O35

**Introduction:** Normal Pressure Hydrocephalus (NPH) patients can significantly benefit from treatment with Ventriculoperitoneal shunts (VPS). There is currently little data on long-term shunt survival in patients with NPH. Patients often ask how long is their shunt is likely to last? This information is important as NPH patients are elderly and can die of unrelated causes. This study describes the survival rates of VPS in NPH.

**Methods:** This is a retrospective single-centre cohort study assessing the shunt survival rates in a consecutive series of NPH patients who had a VPS inserted >10 years ago. Data on shunt survival (months), shunt revisions, complications and mortality was collected from the patients’ electronic records.

**Results:** Forty-seven NPH patients were included (28 males). At the time of VPS insertion, the average age was 77 years (8 SD). Fourteen patients are alive, while 33 died of unrelated causes during the follow-up period after an average time of 77 months (34 SD) from the VPS insertion. Twelve patients required a shunt revision for suspected blockage or for the adjunct of an adjustable gravitational valve. The probability of shunt survival was 98% at 1 year, 89% at 2 years, 79% at 5 years and 40% at 10 years.

**Conclusion:** Despite being elderly, NPH patients have a high probability of shunt survival compared with other hydrocephalic conditions.

**Fig. 1:**
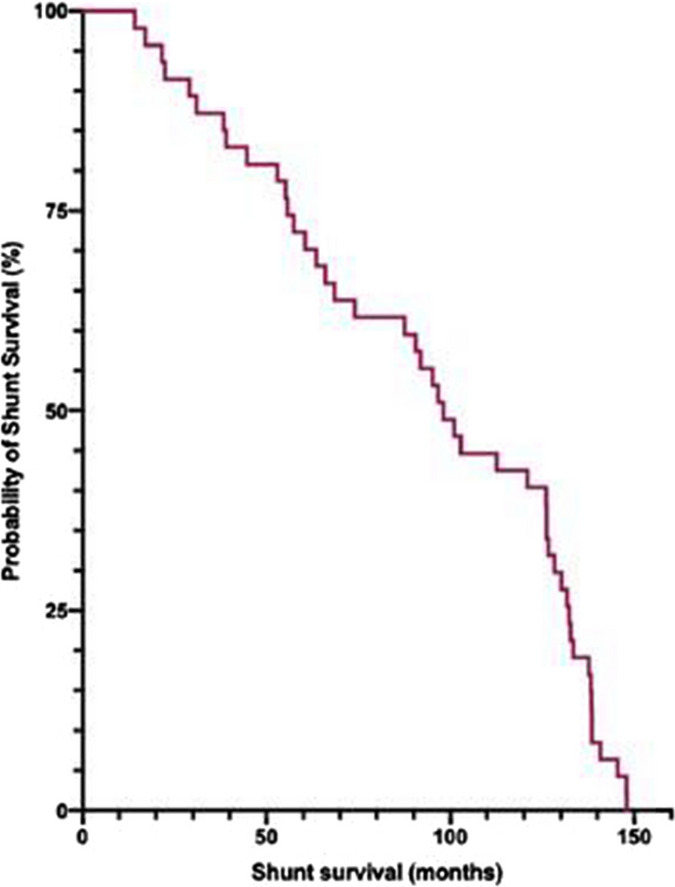
Probability of shunt survival to shunt survival

### O36 Lumbar and ventricular CSF concentrations of extracellular matrix proteins before and after shunt surgery in idiopathic normal pressure hydrocephalus

#### Karolina Minta^1^, Anna Jeppsson^2^, Gunnar Brinkmalm^1,3^, Erik Portelius^1,3^, Henrik Zetterberg^1,3,4,5^, Kaj Blennow^1,3^, Mats Tullberg^2^, Ulf Andreasson^1,3^

##### ^1^Department of Psychiatry and Neurochemistry, Institute of Neuroscience and Physiology, the Sahlgrenska Academy at the University of Gothenburg, Sweden; ^2^Department of Clinical Neuroscience, Institute of Neuroscience and Physiology, the Sahlgrenska Academy at the University of Gothenburg, Sweden; ^3^Clinical Neurochemistry Laboratory, Sahlgrenska University Hospital, Mölndal, Sweden; ^4^Department of Neurodegenerative Disease, UCL Institute of Neurology, London, UK; ^5^UK Dementia Research Institute at UCL, London, UK

###### **Correspondence:** Karolina Minta (karolina.minta@neuro.gu.se)

*Fluids Barriers CNS* 2021, **18(2)**: O36

**Introduction:** Changes in the extracellular matrix (ECM) composition might be involved in the pathophysiology of idiopathic normal pressure hydrocephalus (iNPH). The aim of this study was to explore possible differences between lumbar and ventricular CSF concentrations of the ECM markers brevican and neurocan, matrix metalloproteinases (MMPs) and tissue inhibitor of metalloproteinase-1 (TIMP-1) and their relation to clinical symptoms in iNPH patients before and after shunt surgery.

**Methods:** Paired lumbar and ventricular CSF was collected from 31 iNPH patients, before and four months after shunt surgery. CSF was analysed for concentrations of tryptic peptides originating from brevican and neurocan using a mass spectrometry-based panel, and for MMP- 1, -2, -9, -10 and TIMP-1 using fluorescent or electrochemiluminescent immunoassays.

**Results:** Brevican and neurocan peptide levels were not influenced by CSF origin, but MMP-1, -2, -10 and TIMP-1 were increased (p ≤ 0.0005), and MMP-9 decreased (p ≤ 0.0003) in lumbar CSF compared with ventricular CSF. There was a general trend of ECM proteins to increase following shunt surgery. Ventricular TIMP-1 was inversely correlated with overall symptoms (rho = – 0.62, p < 0.0001).

**Conclusion:** Levels of the CNS-specific proteins brevican and neurocan did not differ between the lumbar and ventricular CSF, whereas the increase of several CNS-unspecific MMPs and TIMP-1 in lumbar CSF suggests contribution from peripheral tissues. The increase of ECM proteins in CSF following shunt surgery could indicate disturbed ECM dynamics in iNPH that are restored by restitution of CSF dynamics.

### O37 Manual pumping of a hydrocephalus shunt: nonsense or a worthwhile diagnostic tool? technical note of non-invasive shunt-testing with video demonstrations

#### Uwe Kehler^1^

##### ^1^Asklepios Klinik Altona

###### **Correspondence:** Uwe Kehler (uwekehler@hotmail.com)

*Fluids Barriers CNS* 2021, **18(2)**: O37

**Introduction:** If a patient is presenting with clinical deterioration after shunt surgery, several diagnostic tools are available to detect the failure and the side of the shunt dysfunction. One possible tool is manual non-invasive shunt pumping, although controversially discussed. Shunt pumping differs technically very much by the valve design and shunt configuration. The technical description, feasibility, indications as well as evaluations will be demonstrated.

**Methods:** Shunt pumping, particularities of different shunt configurations and requirement for successful testing are technically explained and video demonstrated. Also, clinical evaluations are shown.

**Results:** Depending on the hydrocephalus shunt design, evaluation of the shunt function can be more or less easily be done by pumping a reservoir or flushing chamber with simultaneous catheter occlusion by manual compression. With this procedure the detection of occluded catheters (ventricular, peritoneal or the valve itself) is non-invasively possible in a completely outpatients setting. Depending on the shunt design through pumping it is possible to drain a special quantity of CSF - so performing a non-invasive tap-test as well.

**Discussion:** Noninvasive evaluation of shunt failure via pumping is non-invasively with less risks (no infections) and is fast to perform in an outpatient setting. So, pumping can substitute many invasive tests with less risks for the patients (no infections!) and with less time consuming for the medical staff. If the pumping- result will not be conclusive, of course further investigations as imaging and invasive tests with ICP measurement, infusion tests, and others can be added.

### O38 Mathematically designed ventricular catheter, optimized to reduce astrocyte activation through shear reduction and flow redistribution

#### Ahmad Faryami^1^, Christopher Roberts^2^, Dr. Carolyn A. Harris^3^

##### ^1^Biomedical Engineering, Wayne State University, Detroit, Michigan,48202 USA; ^2^Chemical Engineering and Material Science, Wayne State University, Detroit, Michigan,48202 USA; ^3^Chemical Engineering and Material Science, Wayne State University, Detroit, Michigan,48202 USA

###### **Correspondence:** Ahmad Faryami (gw7895@wayne.edu)

*Fluids Barriers CNS* 2021, **18(2)**: O38

**Introduction:** Shunt-associated complications are extremely common with 40% failure within two years of placement. Shunt obstruction accounts for 70% of revisions in the United States. Recent analysis of astrocyte cytokine secretion under shear stress reveal a statistically significant increase in pro-inflammatory IL-6 cytokine secretion. Flow analysis reveals the presence of a shear gradient, inherent to the geometry of commercial catheters, supporting the hypothesis that the catheters need to be optimized to reduce shear.

**Methods:** A CAD model of a ventricular catheter was generated using confocal microscopy for accurate measurements. Computational fluid dynamic modeling was simulated using Ansys Fluent. Using a coupled pressure method, velocity and pressure profiles were extracted into Fluent Post Processing for imaging, revealing increased flow through holes furthest from the catheter tip and localized high shear hotspots.

**Results:** We hypothesized that the observed gradient is the result of uneven fluid velocity inside the lumen, forming a pressure gradient that results in preferential flow through the catheter holes adjacent to regions of high velocity luminal flow. A cone-shaped catheter was generated by applying the ANSYS results to Bernoulli principles. The modified catheter is intentionally identical to control catheters except for its optimized geometry. Ansys analysis of the modified catheter demonstrate uniform flow, pressure, and shear rate throughout the catheter with equal flow through all catheter holes and the elimination of hotspots and shear distribution throughout the catheter.

**Conclusion:** A truly optimized catheter is the implementation of various modifications to current catheters. This is a brief description of catheter geometry optimization.

### O39 CSF fistulas of the skull base: first sign of adult hydrocephalus

#### Rik Demaerel^1,2^, T. Van Havenbergh^1^

##### ^1^Department of Neurosurgery, GZA Sint-Augustinus Antwerp, Belgium; ^2^Department of Neurosurgery, UZ Leuven, Belgium

###### **Correspondence:** Rik Demaerel (rik.demaerel@gmail.com)

*Fluids Barriers CNS* 2021, **18(2)**: O39

**Introduction:** We present three adult patients with a meningoencephalocele in the anterior skull base and rhinorrhea. Secondary and idiopathic increased intracranial pressure (ICP) can cause thinning of cortical bone and can lead to a protrusion of meninges and brain tissue. We present the imaging and surgical treatment in these patients and try to explain the pathophysiology and reflect on the relationship between defects in the skull base and chronic increased ICP in adult patients.

**Material and methods:** Three adult patients presented with rhinorrhea. CT imaging showed bone defects of anterior skull base. MRI revealed a meningoencephalocele and signs of chronic increased ICP (empty sella, narrowing of the aqueduct and disruption of the septum pellucidum). Patients had different causes of the hydrocephalus. The location of the meningoencephalocele was in patient 1 in the ethmoid sinus and lamina cribrosa, in patient 2 in the frontal sinus and lamina cribrosa and in patient 3 in the sphenoid sinus.

**Results:** Patients underwent dual-staged surgery. Endoscopic closure of the fistula was feasible and safe, but turned out not to be enough. The post-operative evolution (recurrence of leakage or symptoms of ICP) shows how these patients require solution of the CSF resorption problem to prevent new leakage.

**Conclusion:** Meningoencephaloceles are probably the consequence of an underlying CSF resorption problem. Chronic increased ICH leads to thinning of the cortical bone. This can lead to a protrusion of brain and meninges. The combination of surgical closure of the leakage and CSF shunting can cure these patients with good outcome on the long term.

### O40 Neuroendoscopic challenges in ventricular tumors treatment

#### Piero A. Oppido^1^

##### ^1^Dpt. of Neurosurgery, IRCCS Regina Elena National Cancer Institute, Rome, Italy

###### **Correspondence:** Piero Andrea Oppido (piero.oppido@ifo.gov.it)

*Fluids Barriers CNS* 2021, **18(2)**: O40

**Introduction:** Frequently, ventricular tumours causing the obstruction of cerebrospinal flow (CSF) pathways, by neuroimaging are documented. Neuroendoscopic procedures enable fenestration of cystic lesions or tumours resection, in addition with third ventriculostomy or septostomy to restore CSF pathways.

**Methods:** In 96 patients, affected by tumours arising by the wall of the third or lateral ventricle, hydrocephalus or obstruction of CSF flow was present. By endoscopic technique, septostomy, cystostomy, third ventriculostomy (ETV) or tumour resection were alone or simultaneously performed to control intracranial hypertension. The (thulium)™ laser for tumours shrinkage and haemostasis of high vascularized tumours was used.

**Results:** In 68 patients with non-communicating hydrocephalus the ETV was realized. In 6 LG astrocytoma the ETV was the only surgical treatment, definitely. In 20 cystic tumours cystostomy and marsupialization into the ventricle solved the mass effect and intracranial hypertension syndrome. In 12 patients neuroendoscopic relief of CSF pathways by septostomy associated to Ommaya reservoir or one catheter shunt was possible. In 6 colloid cysts and 5 cystic craniopharyngiomas removal was possible, by restoring CSF flow without other procedures. After intracranial hypertension control, in 28 malignant gliomas, 18 metastasis or leptomeningeal carcinomatosis and 6 lymphomas tumour adjuvant therapy was performed. In 6 cystic central neurocytomas and 12 ependymomas subsequent microsurgical removal was achieved.

**Conclusion:** In ventricular tumours neuroendoscopy is a challenge but by the Tm laser complications can be reduced. Neuroendoscopy is safe and effective to restore CSF pathways, avoiding major surgical approaches and without any relevant post-operative morbidity.

### O41 Neuroinflammation, white matter and subventricular zone alterations in juvenile pigs with hydrocephalus

#### Maria Garcia-Bonilla^1^, Sarah Zwick^1^, Leandro Castaneyra-Ruiz^1^, Michael Talcott^1,2^, Ayodamola Otun^1^, Albert Isaacs^3^, Diego Morales^1^, David Limbrick Jr.^1^, Pat McAllister^1^

##### ^1^Department of Neurosurgery, Washington University in St. Louis School of Medicine, St. Louis, Missouri, 63110, USA; ^2^Division of Comparative Medicine, Washington University in St. Louis School of Medicine, St. Louis, Missouri, 63110, USA; ^3^Department of Surgery, Division of Neurosurgery, University of Calgary School of Medicine, Calgary, Alberta, T2N 2T9, Canada

###### **Correspondence:** Maria Garcia Bonilla (mariag@wustl.edu)

*Fluids Barriers CNS* 2021, **18(2)**: O41

**Introduction:** Neuropathology in hydrocephalus comprises ventriculomegaly, white matter injury, inflammation, edema, and gliosis in both humans and experimental models. Our group has developed a large animal model of acquired hydrocephalus in juvenile pigs to evaluate the current treatments for the disease. We hypothesized that this pig model mimics the neuropathology found in the periventricular parenchyma described in human hydrocephalic infants.

**Methods:** Hydrocephalus was induced by percutaneous intracisternal kaolin injections in 35- day old pigs (n = 7). Age-matched sham controls received saline injections (n = 6). After 30 days, MRI, immunohistochemistry and cerebrospinal fluid (CSF) protein analyses were performed.

**Results:** The expansion of the ventricles was especially pronounced in the atrium, where ependymal disruption occurred. In this area, the periventricular white matter showed a 44% increase in cell death (p < 0.05) and a 67% reduction of oligodendrocytes (p < 0.01). In the subventricular zone (SVZ), the number of proliferative cells and oligodendrocyte progenitors decreased by 75% and 57% respectively (p < 0.01), suggesting possible neurodevelopment impairment. The decrease of the SVZ area correlated significantly to the ventricular volume increase (p < 0.03). Neuroinflammation occurred in the hydrocephalic pigs with a significant increase of astrocytes and microglia in the white matter (p < 0.02), and high levels of inflammatory interleukins IL-6 and IL-8 in the CSF (p < 0.01).

**Conclusion:** The induction of acquired hydrocephalus produced damage in the periventricular white matter, reduced cell proliferation in the SVZ, and neuroinflammation. These findings mimic those found in human hydrocephalus, demonstrating that the pig model can be a useful tool for preclinical studies of the pathophysiology of hydrocephalus.

### O42 Outcomes associated with a standardized assessment and treatment protocol for 298 patients with suspected idiopathic normal pressure hydrocephalus (iNPH)

#### Mark G Hamilton^1^, Stefan Lang^1^, David Ben-Israel^1^, Nick Sader^1^, Sandeep Muram^1^, Jarred Dronyk^1^, Geberth Urbaneja^1^, Albert Isaacs^1^

##### ^1^Department of Clinical Neurosciences, Division of Neurosurgery, University of Calgary School of Medicine, Calgary, Alberta, Canada

###### **Correspondence:** Mark Hamilton (mghamilton.hydro@gmail.com)

*Fluids Barriers CNS* 2021, **18(2)**: O42

**Objective:** To describe the results of a single-center experience with a standardized assessment and treatment protocol for patients with suspected idiopathic normal pressure hydrocephalus (iNPH).

**Background:** Without an objective assessment protocol to select patients with suspected iNPH for shunt surgery, the shunt response failure rate typically is no higher than 50%.

**Design/methods:** Consecutive patients with suspected iNPH (age> 60 years; ventriculomegaly (Evans’s Index >0.3); gait and cognitive disturbances) presenting between January 2015–July 2020 were evaluated with a standardized protocol that included 10-meter gait velocity (GV), cognitive function (Montreal Cognitive Assessment (MoCA) and Symbol Digits Modalities Test (SDMT). Patients underwent large volume lumbar puncture (LP) or 72-hour external lumbar drainage (ELD) to assess GV and cognitive function pre- and post-LP and ELD. Patients with significant improvement post LP or ELD test were offered treatment with a CSF shunt. Outcome measures were repeated 3-6 months post shunt surgery.

**Results:** 298 patients (183 males; mean age 77 years) were assessed. 91 underwent LP. 41/91 had a negative LP response. 8/41 negative LP patients underwent ELD with a positive response and shunt treatment. GV in positive LP patients increased from 0.62 ± 0.19 to 0.84 ± 0.24 m/sec. 215 patients underwent ELD. 104/215 had a negative ELD response (GV, MoCA and SDMT unchanged). GV in positive ELD patients increased from 0.66 ± 0.35 to 0.88 ± 0.35 m/sec. 164 patients underwent shunt surgery. GV in shunted patients increased from a baseline 0.64 ± 0.31 to 1.05 ± 0.32 m/sec (p = 0.001) at 3 months post shunt while the median MoCA and SDMT increased from 21 to 24 (p = 0.001) and 20 to 25 (p = 0.001), respectively.

**Conclusion:** Using a standardized assessment and treatment protocol for patients with suspected iNPH allows identification of patients with a high probability of significant improvement in gait and cognitive outcomes assessed 3 months after surgery.

### O43 Panventriculomegaly with a wide foramen of magendie and a large cisterna magna (PaVM) – defining the phenotype

#### Adam Nunn^1^, Melissa Werndle^2^, Joao Alves Rosa^2^, Kelly McManus^1^, Rebecca Hodnett^1^, Jack Wildman^1^, William Singleton^1^, Alex Mortimer^2^, Richard Edwards^1^

##### ^1^Department of Neurosurgery, Southmead Hospital, Bristol, UK; ^2^Department of Neuroradiology, Southmead Hospital, Bristol, UK

###### **Correspondence:** Adam Nunn (adam.nunn@nbt.nhs.uk)

*Fluids Barriers CNS* 2021, **18(2)**: O43

**Introduction:** Panventriculomegaly with a wide foramen of Magendie and large cisterna magna (PaVM) describes a type of chronic hydrocephalus that presents with similar clinical features to idiopathic normal pressure hydrocephalus (iNPH).

**Methods:** Patients with PaVM were extracted from an institutional database of 741 consecutive new patients presenting with possible/suspected NPH, following blinded neuroradiology review of referral imaging. All PaVM patients were identified retrospectively (the majority pre- date the description of the condition).

**Results:** We identified 11 cases of PaVM. All underwent ELD and 73% showed improvement. Eight patients underwent permanent CSF diversion (VP shunt) and 75% were improved at 12 months. Compared to the iNPH group (N = 498), PaVM patients had a larger Evans index (0.45 vs. 0.39, P < 0.01) and larger temporal horns (8.4mm vs. 6.6mm, P = 0.02). No difference was seen in callosal angle or convexity tightness, however, narrow Sylvian fissures were more prevalent (55% vs. 1%), as was depressed floor of the third ventricle (50% vs. 0%). None of the non-shunted PaVM patients had a depressed floor of third ventricle. There was a high degree of concordance between tight Sylvian fissures and depressed floor of the third. All those with a depressed floor of third ventricle improved with a shunt.

**Conclusion:** A depressed floor of the third ventricle with a patent aqueduct should prompt consideration of PaVM in patients presenting with NPH. If the floor of third ventricle is depressed, a straight-to-shunt approach is recommended. Improvement with lumbar drainage supports the presence of an intracisternal block as the point of obstruction.

### O44 Parkinsonians signs in inph patients: descriptive and dynamic changes in a prospective cohort of patients

#### Enrico Belgrado^1^, Francesco Tuniz^3^, Yan Thereshko^2^, Daniele Piccolo^3^, Sara Fabbro^3^, Miran Skrap^3^, Daniela Cargnelutti^1^, Gianluigi Gigli^2^

##### ^1^Department of Neurology; ^2^Neurologic Clinic and ^3^Neurosurgery, Azienda Ospedaliero-Universitaria, Santa Maria della Misericordia Udine, Italy

###### **Correspondence:** Francesco Tuniz (tuniz.francesco@gmail.com)

*Fluids Barriers CNS* 2021, **18(2)**: O44

**Introduction:** Parkinsonism is a frequent feature in iNPH patients, but the descriptions are scarce. We used mUPDRS (part III) and classical tools (TUG test, 10MWT, Tinetti, MMSE and FAB) to describe the extrapiramidal features of a prospective cohort of suspected iNPH patients.

**Methods:** 139 consecutive patients suspected having NPH according to International Guidelines were enrolled. Each patient completed a neurological visit before and 24 hours after a lumbar infusion test with tap test (TT). On the basis of shunt effectiveness, clinical and radiological criteria, patients were classified as iNPH, LOVA, NOT-NPH and Probable-NPH.

**Results:** 88% of iNPH, 75% of NOT-NPH, 80% of Probable-NPH and 25% of LOVA have significant (mUPDRS>10 points) extrapyramidal signs; the burden of symptoms is skewed versus the lower body in iNPH (76% have higher scores in lower body) but is present also in the upper limbs (upper bradykinesia score in iNPH equal to NOT-NPH and Probable-NPH). Severity of mUPDRS correlates with TUG, 10MWT and FAB score at baselines. Overall mUPDRS doesn't correlate with Rout neither with age but with duration of symptoms. After TT mUPDRS improves significantly in all except NOT-NPH group. The change (mean 3.3±2.9 points) was higher for iNPH and Probable-NPH and significant versus LOVA and NOT-NPH. Tremor scores didn't change at all as well as score of postural instability. The main contributors to mUPDRS improvement were bradykinesia and some gait scores.

**Conclusion:** extrapyramidal signs are frequent in iNPH patients and they change accordingly to diagnosis after TT; LOVA patients represent a clinical distinct entity.

### O45 Patient generated cerebrospinal fluid reciprocating pump for in-vitro hydrocephalus fluid analysis and long-term cellular response studies

#### Ahmad Faryami^1^, Adam Menkara^2^, Daniel Viar^3^, Dr. Carolyn A. Harris^4^

##### ^1^Biomedical Engineering, Wayne State University, Detroit, Michigan,48202 USA; ^2^Biomedical Engineering, Wayne State University, Detroit, Michigan,48202 USA; ^3^Computer Science and Engineering, University of Toledo, Toledo, Ohio, 43606 USA; ^4^Chemical Engineering and Material Science, Wayne State University, Detroit, Michigan,48202 USA

###### **Correspondence:** Ahmad Faryami (gw7895@wayne.edu)

*Fluids Barriers CNS* 2021, **18(2)**: O45

**Introduction:** The classic presentation of hydrocephalus involves elevated intracranial pressure which manifests with clinical symptoms. The underlying cause of hydrocephalus is not fully understood in many hydrocephalus patients, and recent studies indicate that idiopathic normal pressure hydrocephalus might be more common than it was previously supposed. Therefore, it is imperative to study the CSF production, reabsorption, and its interactions with the cardiovascular system and autonomic nervous system.

**Methods:** A photo-electric pulse monitor and electrocardiography module with a portable data logging device was developed to record patient heart activity in 24-hour cycles. The patient data was transferred to custom-built software for reading raw patient data and generating appropriate output signals for controlling the motors of reciprocating pumps to replicate the pulsations as a function of time. The pump flow rate was set at a constant physiological 0.3 ml/min. The pump output was recorded using a flow sensor.

**Results:** The data logger registered heart rate variability and momentary changes in heart rate. The pump was able to replicate the exact number of recorded pulses, the time interval between two subsequent pulses, while maintaining the constant output volume. Using this setup, an experimental algorithm was also tested to automatically adjust the pump output rate based on the correlations between patient heart output and CSF production rate. The calculated CSF production rate was within the expected physiologic range.

**Conclusion:** A recording and simulation in-vitro setup was developed to study the impact of pulsating flow on immune cells and shunt system.

### O46 Predicting shunt success after perinatal post-haemorrhagic hydrocephalus

#### Saeed Kayhanian^1^, Jonathan P Funnell^1^, Katharina P Zühlsdorff^2^, Rory J Piper^3^, Timothy P Lawrence^3^, Ibrahim Jalloh^1^

##### ^1^Division of Neurosurgery, Department of Clinical Neurosciences, University of Cambridge, UK; ^2^Department of Psychology, University of Cambridge, UK; ^3^Department of Neurosurgery, Oxford University Hospitals, UK

###### **Correspondence:** Saeed Kayhanian (saeedkay90@hotmail.com)

*Fluids Barriers CNS* 2021, **18(2)**: O46

**Introduction:** Intra-ventricular haemorrhage (IVH) is common in premature neonates. An estimated 15% of neonates who suffer IVH will develop post-haemorrhagic hydrocephalus (PHH) requiring permanent CSF diversion. Management of perinatal PHH may begin with temporising measures (e.g. with a ventricular access device) to reduce ventricular CSF volume. Definitive CSF diversion is often delayed until the infant is older – but there is no consensus or guideline on timing. It has been suggested that the delayed insertion of a permanent shunt is associated with fewer infections and shunt failures. The aim of this study was to identify factors associated with shunt success in this cohort.

**Methods:** Single-centre retrospective review of operative records between 2015-2019, to identify patients undergoing primary shunt insertion for PHH. Clinical characteristics (including gestational age at birth and shunt, Papile grade of IVH, weight, occipital-frontal circumference) and causes for any shunt failure were extracted from electronic patient records. A generalised linear model was constructed to fit the dichotomised outcome of shunt success or failure at 12 months.

**Results:** 26 patients (16 male) underwent ventriculo-peritoneal shunt insertion for PHH in this period. 10 patients suffered from shunt failure within the first 12 months. The most common causes of failure were migration of the proximal catheter (n = 4), infection (n = 2), and obstruction (n = 2). Weight at the time of shunt insertion was highly predictive for shunt success (F = 6.6, p = 0.02), with no other characteristic significantly correlated.

**Conclusion:** Careful stratification by weight may improve outcomes for shunting in infants with PHH. Larger cohort studies are planned to confirm this finding and we intend to use these to derive a multi-factorial PHH shunt success score.

### O47 Prediction of outcome after shunt surgery through csf biomarkers – the point-CSF Study

#### H Sabir^1^, A Jeppsson^1^, K Andrén^1^, K Laurell^2^, H Zetterberg^3,4^, K Blennow^3^, C Wikkelsø^1^, P Hellstrom^1^, M Tullberg^1^

##### ^1^Hydrocephalus research unit, Department of Clinical Neuroscience, Institute of Neuroscience and Physiology, Sahlgrenska Academy, University of Gothenburg, Sweden; ^2^Department of Neuroscience, Uppsala University, , Sweden; ^3^Clinical Neurochemistry Laboratory, Sahlgrenska University Hospital, Mölndal, Sweden, Department of Psychiatry and Neurochemistry, Institute of Neuroscience and Physiology, Sahlgrenska Academy, University of Gothenburg, Sweden; ^4^Department of Neurodegenerative Disease, UCL Institute of Neurology, Queen Square, London, UK and UK Dementia Research Institute at UCL, London, UK

###### **Correspondence:** Hemin Sabir (hemin.sabir@vgregion.se)

*Fluids Barriers CNS* 2021, **18(2)**: O47

**Introduction:** Shunt surgery is an effective treatment in idiopathic normal pressure hydrocephalus (iNPH). Robust preoperative predictors of the effect of shunting are however lacking. Several CSF biomarkers are available that each reflects a specific pathophysiological process in the brain. The POiNT-CSF study (Prediction of Outcome in iNPH through CSF biomarkers) comprise a cohort of prospectively evaluated shunt operated iNPH patients with the aim of identifying predictive biomarkers for improvement

**Aim:** To identify patterns of CSF-biomarkers that can predict a significant clinical improvement after shunt surgery as measured by the iNPH-scale.

**Methods:** One-hundred and fourty consecutive patients shunt operated for iNPH were included. Clinical symptoms were scored pre- and 4 months postoperatively on the iNPH scale and improvement defined as ≥ 5 points postoperative increase. Preoperative lumbar CSF was analysed for Aβ40, Aβ42 and the Aβ40/Aβ42 ratio; soluble amyloid precursor proteins (sAPPα and sAPPβ), total and phosphorylated tau protein (T-tau and P-tau, respectively), neurofilament light chain (NFL) and glial fibrillary acidic protein (GFAP).

**Results:** Seventy-three percent of the patients had improved at the postoperative evaluation. Levels of sAPPβ and P-tau were significantly higher in unimproved patients compared to improved (p < 0.05) whereas sAPPα level was higher at trend level (p = 0.05). No biomarker correlated with the degree of clinical improvement.

**Conclusion:** None of the studied CSF biomarkers showed a robust association to outcome after shunting. The higher levels of P-tau and sAPPα in unimproved patients indicate that a comorbid pathology such as Alzheimer’s disease could influence the degree of improvement.

### O48 Prediction of outcome in iNPH through clinical signs and symptoms (point-c): a prospective study of 143 patients

#### Kerstin Andrén^1^, Lena Kollén^1^, Anna Jeppsson^1^, Simon Agerskov^1^, Daniel Jaraj^1^, Dan Farahmand^1^, Doerthe Ziegelitz^2^, Mats Tullberg^1^, Per Hellström^1^**contributed equally

##### ^1^Hydrocephalus Research Unit, Institute of Neuroscience and Physiology, Department of Clinical Neuroscience, The Sahlgrenska Academy, University of Gothenburg, Sweden; ^2^Department of Radiology, Institute of Clinical Sciences, The Sahlgrenska Academy, University of Gothenburg, and Sahlgrenska University Hospital, Gothenburg, Sweden

###### **Correspondence:** Kerstin Andren (kerstin.andren@vgregion.se)

*Fluids Barriers CNS* 2021, **18(2)**: O48

**Introduction:** In modern studies, around 80% of treated patients with idiopathic normal pressure hydrocephalus, iNPH, improve in their symptoms. To date there is no test that can reliably predict which patients will benefit from shunt surgery. This study aims to explore the predictive value of symptoms and signs in the clinical picture of iNPH.

**Methods:** A prospective dual-centre study, with inclusion of all patients diagnosed with iNPH October 2014 to June 2016, who underwent shunt surgery and in whom postoperative assessment of iNPH symptoms were possible, n = 143. Clinical data were collected pre- and median 5 months postoperatively. Logistic regression analyses were used to assess outcome defined as improvement or not (by ≥5 points) in the iNPH scale.

**Results:** After surgery, 73% of the patients were improved. Each of the symptom domains as well as the total iNPH scale score improved significantly (median 53 to 69, p < 0.001). The proportions of patients with the following clinical signs decreased significantly: shuffling gait, broad-based gait, paratonic rigidity and retropulsion. A range of gait, mobility and balance tests were all significantly improved, and patients slept significantly shorter postoperatively. Univariable logistic regression analyses of all baseline clinical variables, did not yield any predictors of beneficial outcome with a significance level of <0.10.

**Conclusion:** The study confirms that the recorded clinical signs, symptoms, and impairments in the adopted clinical tests are characteristic findings in iNPH, as they all improved after shunt surgery. However, these clinical data cannot predict which patients will benefit from shunt surgery.

### O49 Prevalence of fecal incontinence in normal pressure hydrocephalus (NPH) patients

#### Uwe Kehler^1^, Sven Petersen^1^

##### ^1^Asklepios Klinik Altona

###### **Correspondence:** Uwe Kehler (uwekehler@hotmail.com)

*Fluids Barriers CNS* 2021, **18(2)**: O49

**Introduction:** Bladder dysfunction is one of the main symptoms of NPH beside gait disturbance and cognitive decline forming the Hakim Triad. Fecal urgency and incontinence is often described as an additional symptom, however, no exact numbers are found in the literature. The aim of this study was to investigate the prevalence of fecal urgency and incontinence in NPH patients.

**Methods and patients:** All patients who presented to our outpatient department or presented to shunt surgery since Jan. 2021 with confirmed diagnosis of NPH were interviewed about fecal function. Additionally, the extent of gait disturbance, cognitive decline, ventriculomegaly (Evans-Index), DESH presence, age, gender and length of history were documented to investigate if there might be any interrelated dependence.

**Results:** 50 patients were evaluated (35 men, 15 females, medium age 77.1 years, median length of history of NPH: 3,26 years, median Evans Index: 0,37, 43 with positive DESH pattern). 48 patients showed gait disturbance, 41 a cognitive decline and 39 bladder dysfunctions. 34 (68%) patients showed the complete Hakim triad. 19 (38%) patients complained about fecal incontinence (14 with urge incontinence, 5 with complete incontinence). There was no clear dependence between fecal disturbance and sex, age, length of history, but with dementia.

**Discussion/conclusion:** Fecal urgency and incontinence is a frequent finding in NPH (38%) and is essential for the quality of live. In the general population, fecal incontinence in elderly is found up to 15%. The more than twofold higher prevalence in NPH patients suggests that NPH causes directly fecal disturbance in a larger percentage.

### O50 Pro-hydro-ger: a geriatric approach to define complexity and frailty in inph patients

#### Liliana Mazza^1^, Aldina Gardellini^1^, Sevil Yaşar^2^, David Milletti^3^, Giulia Giannini^4^, Giorgio Palandri^5^

##### ^1^UOC Geriatria, Ospedale Maggiore, AUSL di Bologna, Bologna, Italia; ^2^Johns Hopkins School of Medicine, Baltimore, U.S.A.; ^3^IRCCS Istituto delle Scienze Neurologiche, UOSI Medicina Riabilitativa, Bologna, Italia; ^4^IRCCS Istituto delle Scienze Neurologiche di Bologna, Clinica Neurologica Metropolitana NEUROMET, Bologna, Italia; ^5^IRCSS Istituto delle Scienze Neurologiche di Bologna, UOC Neurochirurgia, Bologna, Italia

###### **Correspondence:** Liliana Mazza: (liliana.mazza3@gmail.com)

*Fluids Barriers CNS* 2021, **18(2)**: O50

**Introduction:** Idiopathic Normal Pressure Hydrocephalus (iNPH) is a syndrome described by the symptomatic triad of dysfunction, cognitive impairment, gait and urinary incontinence. The diagnosis is based on ventricular dilation and clinical symptoms with normal intracranial pressure. The role of clinical interpretation in iNPH remains crucial, as multiple comorbidities may affect iNPH patients, which is typical of elderly people. In this context, Geriatrician may represent an equally important role to the neurosurgeon in the evaluation of complex and frail patients which may affect surgical options, timeliness and effectiveness.

**Methods:** Comprehensive Geriatric Assessment (CGA) will be applied to iNPH patients. The evaluation of social, functional, clinical and cognitive domains will be performed using various screening scores described in the literature. Focus will be on global assessment of frailty and mortality risk. The disease impact on caregivers will be investigated by Caregiver Burden Inventory (CBI). Patients will be followed at 6- and 12-months.

**Results:** CGA will guide treatment pathways, because suspected iNPH patients will be screened and risk stratified. This way, we expect a better selection of patients for surgical procedure with decreased intra- and post-operative complications. And at the same time, we also expect better surgical outcomes at follow-up months 6 and 12.

**Conclusion:** The presence of Geriatricians in multidisciplinary teams dedicated to surgical patients is well established in the current literature. Still, collaborative work between neurosurgery and geriatric is just recently arising. The geriatric assessment in iNPH patients could be beneficial and innovative for the co-management of frail and complex patients.

### O51 Quality of life and depressive symptoms in idiopathic normal pressure hydrocephalus: reports from a population-based study

#### Andersson J, MD^1^; Maripuu M, MD,PhD^1^; Sjövill M,,MD^1^; Lindam A, MSc^2^; Laurell K, MD, PhD^3^

##### ^1^Department of Clinical Sciences, Umeå University, Sweden; ^2^Department of Public Health and Clinical Medicine, Unit of Research, Education and Development Östersund Hospital, Umeå University, Umeå, Sweden; ^3^Department of Neuroscience, Neurology, Uppsala University, Sweden

###### **Correspondence:** Katarina Laurell (katarina.laurell@neuro.uu.se)

*Fluids Barriers CNS* 2021, **18(2)**: O51

**Objectives:** There are only a limited number of reports on self-rated quality of life and symptoms of depression in idiopathic normal pressure hydrocephalus, especially from a population-based perspective. The objective was to compare health related quality of life (HRQoL) and depressive symptoms between individuals with and without idiopathic normal pressure hydrocephalus (iNPH).

**Methods:** A total of 122 individuals from the general population (30 with iNPH), median age 75 years, 67 females, underwent neurological examinations and computed tomography of the brain with standardised rating of imaging findings and clinical symptoms. The participants completed the Geriatric Depression Scale (GDS-15) and the HRQoL instrument EQ5D-5L. The sample derived from the general population, which diminish the risk of inclusion bias.

**Results:** Participants with iNPH reported a higher median score on GDS-15 (Md = 3) than those with unlikely iNPH (Md = 1) (p < 0.05). Further, those with iNPH rated their HRQoL lower (VAS-scale = 70, EQ5D-5L index = 0.79) than those without (VAS-scale = 80, EQ5D-5L index = 0.86) (p < 0.05).

**Conclusion:** Individuals with iNPH reported more depressive symptoms and a lower HRQoL than those without the condition, underlining the need for shunt surgery as this treatment has been reported to improve HRQoL in previous studies.

### O52 Real-time non-invasive monitoring of cerebrospinal fluid shunt flow

#### Hany M Arafa^1^, Constantine L Karras^2^, Nikhil K Murthy^2^, Collin J Larkin^2^, Amit B Ayer^3^, Siddharth R Krishnan^4^, John A Rogers^1^, Matthew B Potts^2^

##### ^1^Center for Bio-Integrated Electronics, Department of Biomedical Engineering, McCormick School of Engineering, Northwestern University, Evanston, IL 60208; ^2^Department of Neurological Surgery, Feinberg School of Medicine, Northwestern University, Chicago, IL 60611; ^3^Department of Neurological Surgery, Stanford University School of Medicine, Stanford, CA 94304; ^4^Koch Institute for Integrative Cancer Research, Massachusetts Institute of Technology, Cambridge, MA 02139; Departments of Anesthesiology, Critical Care, and Pain Medicine, Boston Children’s Hospital, Boston, MA 02115

###### **Correspondence:** Matthew Potts (matthew.potts@northwestern.edu)

*Fluids Barriers CNS* 2021, **18(2)**: O52

**Introduction:** Current cerebrospinal fluid (CSF) shunts do not allow for real-time monitoring of shunt flow. The hydrodynamics of shunted hydrocephalus during normal activities are therefore poorly understood. Recent technology allows for real-time non-invasive measurement of flow through an implanted shunt by measuring temperature difference upstream and downstream of a thermal actuator. We sought to use this technology to measure shunt flow in real-time during normal position changes.

**Methods:** CSF flow through implanted shunts was monitored using a non-invasive, wearable flow sensor placed on the skin overlying the distal shunt catheter just above the clavicle. Patients started in a supine position and were then transitioned to a sitting position during monitoring. Shunt flow was assessed both qualitatively and quantitatively. Basic patient demographics (age, hydrocephalus etiology, shunt valve and setting) were also recorded.

**Results:** Real-time CSF flow measurements were performed in 15 adult patients. Mean age was 57.3 years (range 18-80 years). Hydrocephalus etiology included normal pressure hydrocephalus (8 patients), tumor (2), idiopathic intracranial hypertension (2), trauma (1), hemorrhage (1), and congenital (1). All patients had programmable shunt valves. Shunt flow was demonstrated in all cases and increased from supine to sitting in all but one patient (p < 0.0001).

**Conclusion:** Real-time non-invasive measurement of CSF flow through an implanted shunt shows that flow changes in a significant and measurable way when patients transition from a supine to a sitting position. Further studies demonstrating real-time shunt flow will allow for a better understanding of the hydrodynamics of shunted hydrocephalus.

### O53 Revert—an EU project to establish a clinical network for transforming the current management of NPH in the UK and France

#### Peter Smielewski^1^, Olivier Baledent^2^, Alexis Joannides^1,3^, Cyrille Capel^4^, Yael Fregier^5^, Michael Martin^6^, Zofia Czosnyka^1,2^, Olivier Peltre^5^, Romuald Seizeur^7^, Alin Bohra^8^, Matthew Garnett^3^, Marija Drinjakovic^1^, Marek Czosnyka^1,2^

##### ^1^Brain Physics Laboratory, Department of Clinical Neurosciences, University of Cambridge, Cambridge UK; ^2^CHIMERE Laboratory, University of Picardie Jules Verne, Amiens, France; ^3^Division of Neurosurgery, Cambridge University Hospitals, Cambridge Biomedical Campus, Cambridge, UK; ^4^Neurosurgery Dept, University Hospital of Amiens, Amiens, France; ^5^Lens Mathematics Laboratory, University of Artois, Arras, France; ^6^Obex Technologies, Cambridge, UK; ^7^Neurosurgery Dept, University Hospital of Brest, Brest, France; ^8^Neurosurgery Dept, University Hospital of Caen, Caen, France

###### **Correspondence:** Peter Smielewski (ps10011@cam.ac.uk)

*Fluids Barriers CNS* 2021, **18(2)**: O53

**Introduction:** Normal Pressure Hydrocephalus (NPH) remains a diagnostic and therapeutic dilemma. Accurate and timely surgical management can lead to considerable improvement in disease course and quality of life. However, identification of patients with NPH who are likely to respond to surgical treatment is challenging, particularly in the elderly who may have multiple concurrent co-morbidities. Furthermore, there are no consensus guidelines to date for optimal NPH management.

**Methods:** The ‘REVERsible dementia projecT (REVERT) aims to improve clinical diagnosis and management of NPH in the UK-French cross-border region through a combined approach of establishing a common clinical network of excellence to transform the current management pathway, clinical informatics, and the parallel development of novel diagnostic tools based on CSF Infusion test and PC MRI pulse-flow morphology analysis. REVERT is a collaboration involving a consortium of clinicians, physicists, mathematicians and software specialists.

**Results:** REVERT was approved by the European Regional Development Fund via the Interreg France (Channel) England Programme in October 2020 and is currently underway.

**Conclusion:** The ultimate objective of REVERT is to work towards a unique solution consisting of a combined diagnostics approach including analysis and interpretation of PCMRI images (pulse morphology analysis) and CSF Infusion tests, with an underlying AI model to guide diagnosis based on both flow and pressure measurements and Web portal for streamlining referrals, patient management, diagnostic/clinical results and including audit tools of standard of care evaluation (also linked to the national shunt registry and the shunt evaluation registry).

### O54 Squire: a novel multi-dye in vivo system for testing real-time neuroinflammation during shunting

#### Jeffrey Horbatiuk^1^, Carolyn A Harris, PhD^2,3,4^

##### ^1^Department of Chemistry, Wayne State University, Detroit, USA; ^2^Department of Chemical Engineering, Wayne State University, Detroit, USA; ^3^Department of Biomedical Engineering, Wayne State University, Detroit, USA; ^4^Department of Neurosurgery, Wayne State University, Detroit, USA

###### **Correspondence:** Jeffrey Horbatiuk (jeff.horbatiuk@gmail.com)

*Fluids Barriers CNS* 2021, **18(2)**: O54

**Introduction:** Recent post hoc studies have shown shunt obstruction is caused by a predominately astrocytic ingrowth, with a small percent of catheters obstructed with choroid plexus. However, the catalysts remain a mystery. Currently, real time in vivo studies exist but do not study more than two cell types at a time. To develop better treatments, multifactorial models need to be a priority. Here, we present a new model system showing cell interplay as a function of shunt insertion speed.

**Methods:** Cell-permanent dyes staining for astrocytes (Sulforhodamine 101), microglia (Lycopersicon esculentum tomato lectin), neurons (4-Chlorobenzenesulfonate Salt), bloodborne macrophages (F4/80), and calcium (Cal-520) were either injected into the venous system or directly into the cortex of adult Sprague-Dawley rats. These dyes were chosen to validate the system because they are part of the well documented tripartite synapse. 1, 5, and 10 mm/s. 10x confocal microscope images recorded at speeds up to 25 frames per second, were stitched together could track the cell’s movement and communication.

**Results:** This model shows that live simultaneous labeling can be assayed and suggests that different insertion speeds lead to different immune responses. Future work will create a repeated measures in vivo system during which changes in cellular response can be documented.

**Conclusion:** By looking at the initial neuroinflammation response, better shunt coatings can be designed using time release technologies. While some studies have investigated these propositions in pieces, this is among the first models to simultaneously test each mechanism in addition as a function of shunting speed.

### O55 The first case of communicating hydrocephalus treated with an endovascular cerebrospinal fluid shunt

#### C. Heilman, M.D., A. Malek, M.D., Ph.D., P. Lylyk, M.D., I. Lylyk, M.D., C. Bleise, M.D., E. Scrivano, M.D., P. Lylyk, M.D.

##### ^1^Division of Neurosurgery, ENERI Medical Institute, Equipo de Neurocirugia Endovascular y Radiologia Intervencionista, Buenos Aires, Argentina PL, IL, CB, ES, PL), Department of Neurosurgery, Tufts Medical Center, Boston, MA 02111 USA (CH, AM)

###### **Correspondence:** Adel Malek (clipandcoil@gmail.com)

*Fluids Barriers CNS* 2021, **18(2)**: O55

**Introduction:** A new cerebrospinal fluid (CSF) shunt device (CereVasc eShunt) has been developed for insertion by a percutaneous transvenous endovascular approach. A clinical trial in patients with post-subarachnoid hemorrhage (SAH) hydrocephalus is underway. We present here our clinical experience with the first patient treated with this device.

**Methods:** An 84-year-old woman with SAH underwent aneurysm coiling. An external ventricular drain (EVD) was placed for communicating hydrocephalus. An EVD clamp trial on day 9 showed intracranial pressure (ICP) of 44 cmH2O. On day 10 she underwent endovascular placement of the eShunt device from the cerebello-pontine angle (CPA) cistern to the jugular vein.

**Results:** The patient’s EVD was closed eight hours prior to the procedure. ICP immediately prior to implant deployment was 38 cmH2O. Following implant placement, the ICP reached normal levels (< 20 cmH2O) within 90 minutes. A post-implant CT scan showed no blood in the CPA cistern. The patient’s ICP was monitored through the EVD for 39 hours post-procedure at which point the EVD was safely removed. An MRI six days post-procedure showed reduction in the size of the lateral and third ventricles.

**Discussion:** We describe the first patient treated for communicating hydrocephalus using the novel eShunt endovascular CSF diversion implant. Following device deployment, the patient experienced a rapid reduction of ICP to normal levels (< 20 cmH_2_O), coupled with a reduction in ventricular size. No bleeding or adverse effects occurred during the three-week follow-up period. This is the first patient treated for communicating hydrocephalus by an endovascular approach without the need for a burr hole, brain penetration or multiple skin incisions.

**Declarations**: The authors declare that the patient has give written consent for the publication of this study.

### O56 The value of pre-shunt CSF biomarkers in idiopathic normal pressure hydrocephalus: does it matter?

#### R. Gambin^1^, A. Musumeci^1^, M. Testa, G. Zanusso^2^, F. Cozzi, F. Sala^1^, G. Pinna^1^

##### ^1^Department of Neuroscience, Institute of Neurosurgery, Azienda Ospedaliera Universitaria Integrata di Verona, Verona, Italy; ^2^Department of Neuroscience, Neurological Clinic, University of Verona, Verona, Italy

###### **Correspondence:** Roberta Gambin (roberta.gambin@aovr.veneto.it)

*Fluids Barriers CNS* 2021, **18(2)**: O56

**Introduction:** iNPH is characterized by Hakim triad. Unclear pathogenesis leads to abnormal dynamics of CSF and secondary neurodegeneration that can be co-existing and participate secondary or primarily in the genesis of ventricular enlargement. The aim of our work was to assess whether there was a subgroup not susceptible to shunt, with clinical and liquor biomarkers, indices of progressive dementia: T-tau, P-tau181, Abeta42, Abeta42/P-tau181 ratio.

**Methods:** 52 patients with suspected iNPH were assessed for TAP test; MMSE and walking test, prior and after 2/72 hours CSF removal. CT/MRI showed dilated ventricles, Evans’ ratio>0.3. We removed 30mL of CSF. We analyzed CFS concentration (ng/L) of Abeta42, P-tau181, T-tau, ratio P-tau181/Abeta42 (ELISA) to address surgical treatment.

**Results:** Mean age 76 years. All with Hakim triad. Mean MMSE 23; 31 patient had prevalence walking disorder (group A), 21 increased cognitive difficulties (group B). In group A, T-tau 224, P-tau181 32.8, Abeta42 599, ratio 21. In group B, T-tau 360 rises, Abeta42 489 decreases, P-tau181 38.4, ratio 14.7 up to 17.7, cut-off for progression to Alzheimer (Hansson). 24 patients (> with gait disturbance) improved at the TAP test, were shunted.

**Conclusion:** iNPH is a neurodegenerative condition reversible with surgery. CFS biomarkers analysis helps to better identify tap test no responders. The rate of group B for T-tau and Abeta42, Hazard ratio 14.7 can be index of risk of progression to dementia. We believe that liquor biomarkers in iNPH can be useful for better selection of patients who can be shunted for long- term improvement.

### O57 Transorbital ultrasound and lumbar infusion test in idiopatic intracranial hypertension

#### Francesco Tuniz^2^, Enrico Belgrado^1^, Simone Lorenzut^1^, Fabbro Sara^2^, Daniele Piccolo^2^, ElisaGarbin^2^, Miran Skrap^2^

##### ^1^Department of Neurology and 2 Neurosurgery, ASUFC Azienda Sanitaria Universitaria Friuli Centrale, Santa Maria della Misericordia Udine, Italy

###### **Correspondence:** Francesco Tuniz (tuniz.francesco@gmail.com)

*Fluids Barriers CNS* 2021, **18(2)**: O57

**Introduction:** Optic nerve sheath diameter (ONSD) can be used to estimate intracranial pressure in a non-invasive way. The aim of our study was to evaluate the changing of ONSD during lumbar infusion test and tap test as a tool to guide the effectiveness of the CSF withdraw and the induced decrease of in intracranial pressure. We continuously measured the sub- arachnoid pressure during the procedure correlating with ONSD.

**Methods:** we enrolled a series of patients clinically affected by possible IIH. We performed a lumbar CSF pressure monitoring, infusion test and tap test with continuous ultrasound measurement of ONSD with a 7,5 Mhz linear probe. We performed a CSF tap according to real- time OSND reduction. We measured lumbar subarachnoid pressures in all the patients with LiquoGuard 7 ® device.

**Results:** we studied 6 patients with IIH. After the tap test we obtained a significant reduction of ONSD of 0,875 mm (range 0,6-1 mm) in all patients. All the patients performed a pre and post- test ophtalmological evaluation with OCT. A case of asymmetric papilledema was reported. All patient except one showed improvement of symptoms after the procedure.

**Conclusion:** continuous ultrasound measurement of ONSD can became an important bed- side instrument to guide the correct execution of lumbar CSF pressure monitoring, infusion test and tap test in IIH. Moreover, transorbital ultrasound can be easily used as a follow up tool in IIH.

### O58 Upper limb motor impairment in idiopathic normal pressure hydrocephalus. a cross sectional descriptive study

#### Magelli Elena^1^, David Milletti^1^, Giorgio Palandri^2^, Paolo Mantovani^2^, Giulia Giannini^3^, Sabina Cevoli^3^, Luca Albini Riccioli^4^, Liliana Mazza^5^, Pietro Cortelli

##### ^1^UOSI Medicina Riabilitativa, IRCCS Istituto delle Scienze Neurologiche, Bologna, Italy; ^2^UOC Neurochirurgia, IRCSS Istituto delle Scienze Neurologiche di Bologna, Bologna, Italy; ^3^Clinica Neurologica Metropolitana NEUROMET, IRCCS Istituto delle Scienze Neurologiche, Bologna, Italy; ^4^UOC Neuroradiologia, IRCCS Istituto delle Scienze Neurologiche di Bologna, Bologna, Italy; ^5^UOC Geriatria, Ospedale Maggiore, AUSL di Bologna, Bologna, Italy

###### **Correspondence:** Elena Magelli (elena.magelli@ausl.bologna.it)

*Fluids Barriers CNS* 2021, **18(2)**: O58

**Introduction:** Idiopathic normal pressure hydrocephalus (iNPH) is an elderly-onset syndrome characterised by progressive cognitive impairment, gait apraxia and urinary incontinence. The diagnosis is based on chronic ventricular dilation associated with normal cerebrospinal fluid pressure. Despite gait alterations are broadly studied in literature, a lack of emphasis is commonly placed on possible impairment involving other motor functions, such as those related to the upper limb. The aim of the study was to investigate and describe upper limb motor alterations in iNPH.

**Methods:** A cohort of iNPH’s patients and a control group of healthy subjects, underwent upper limb evaluation of ideomotor apraxia (Spinner-Tognoni’s Test), executive functions (Luria’s Test), bradykinesia and tremor, manual dexterity (Nine Holes Peg’s test). A descriptive statistics was conducted; Fisher Yates-Test was used to analyse statistically significative difference between groups.

**Results:** A total of 49 subjects were recruited (30 iNPH patients, 19 controls). Groups were homogeneous in terms of demographic characteristics and sex. Data analysis showed a significative alteration of upper limb motor function in iNPH in terms of motor sequences organisation (Luria’s Test, p value < 0.0001), bradykinesia and impaired dexterity. Ideomotor apraxia was not present (Spinner-Tognoni’s Test, p value = 1).

**Conclusion:** Apraxic and extrapyramidal features may involve upper limb’s function and ability in daily activities. In particular, impairment of executive functions in terms of organisation and production of motor sequences, may affect the ability to perform complex motor tasks as postural transitions or walking with aids.

### 059 Very rare ventricular arachnoid cysts in adults mimicking NPH syndrome

#### Piero A. Oppido^1^

##### ^1^Dpt. of Neurosurgery, IRCCS Regina Elena National Cancer Institute, Rome, Italy

###### **Correspondence:** Piero Andrea Oppido (piero.oppido@ifo.gov.it)

*Fluids Barriers CNS* 2021, **18(2)**: O59

**Introduction:** The intracranial arachnoid cysts are 1% of expanding lesions in pediatric age, but very rare in adults. Especially, in the ventricular system the cerebrospinal flow (CSF) can be obstructed, by developing hydrocephalus. The endoscopic treatment with cystostomy and combined septostomy or ETV can restore the CSF pathways.

**Methods:** Seven adult patients (age 27 -67 yrs.) were affected by ventricular arachnoid cysts. The site was: occipital horn, septum pellucidum, 3° and 4° ventricle, mesencephalic cistern, vermis of cerebellum. At admission, all patients complained with gait imbalance and signs of chronic intracranial hypertension, as the NPH syndrome. A flexible or rigid endoscope and in 4 cases the Tm laser (LISA) were used.

**Results:** In 6 cases the cystostomy was performed, while in 1 only the ETV. In 2 cases combined cystostomy and ETV or septostomy were achieved. In 2 cases by Tm laser the cyst’s wall was almost completely vaporized to get a full communication with the CSF pathways. The surgical median time was 65 minutes. After endoscopic procedure, all patients improved with complete recovery. No postoperative morbidity or mortality. At follow-up (from 2 to 7 years) all patients have still a good quality of life and normal social activity.

**Conclusion:** The endoscopic treatment of intracranial arachnoid cysts is a safe and mininvasive surgical procedure without complicances and long follow-up good results. In adults mimicking NPH syndrome, neuroendoscopy restores CSF pathways achieving quick recovery. The Tm laser was helpful to remove the cyst’s wall for wide communication into the ventricular system.

### O60 Pilot study of a multi-center, randomized controlled trial of shunt surgery in iNPH

#### Adult Hydrocephalus Clinical Research Network: Jan Malm^9^, Mark G. Hamilton^1^, Rich Holubkov^2^, Sean Nagel^3^, Jeffery Wisoff^4^, Guy McKhann^5^, Tom Zwimpfer^6^, Richard Edwards^7^, Abhay Moghekar^10^, Anders Eklund^9^, James Golomb^4^, Heather Katzen^11^, Nick Dasher^8^, Michael A. Williams^8^, Mark Luciano^10^

##### ^1^University of Calgary, Calgary, AB, Canada; ^2^University of Utah, Salt Lake City, UT, USA; ^3^Cleveland Clinic Foundation, Cleveland, OH, USA; ^4^NYU Langone Health, New York, NY, USA; ^5^Columbia University School of Medicine, New York, NY, USA; ^6^University of British Columbia, Vancouver, BC, Canada; ^7^University of Bristol, Bristol, United Kingdom; ^8^University of Washington, Seattle, WA, USA; ^9^Umea University, Umea, Sweden; ^10^John Hopkins School of Medicine, Baltimore, MD, USA; ^11^University of Miami, Miami, FL, USA

###### **Correspondence:** Jan Malm, Dep Clinical Science, Neurosciences, Umeå University, Sweden (jan.malm@umu.se)

*Fluids Barriers CNS* 2021, **18(2)**: O60

**Background:** To describe preliminary results of a multi-center, randomized, blinded, placebo- controlled, pilot trial of shunt surgery in INPH.

**Methods:** Five sites randomized 18 patients scheduled for ventriculoperitoneal shunting based on CSF-drainage response. Patients were randomized to a Codman® Certas® Plus valve with SiphonGuard at either setting 4 (Active, N = 9) or setting 8/”virtual off” (Placebo, N = 9). Patients and assessors were blinded to the shunt setting. Outcomes included 10-meter gait velocity, cognitive function, and bladder activity scores. The prespecified primary analysis compared changes in 4-month gait velocity in the Active versus Placebo groups. After the 4 months follow up, all shunts were opened, i.e., adjusted to setting 4 whereafter patients underwent 8 and 12-month post-surgical assessment. At the 8-month follow-up, the Placebo group had had an open shunt for 4 months and the Active group for 8 months.

**Results:** At 4-months, gait velocity increased by 0.28±0.28m/s in the Active Group and 0.04±0.17m/s in the Placebo Group (p = 0.071). Overactive Bladder (OAB-q) scores improved in the Active versus Placebo groups (p = 0.007). At 8 months, Placebo gait velocity increased by 0.36±0.27m/s and was comparable to the Active Group (0.40±0.20m/s; p = 0.56).

**Conclusions:** This study shows a trend suggesting gait velocity improves more at an Active shunt setting than a Placebo shunt setting and demonstrates the feasibility of a placebo- controlled trial in iNPH.

## Abstracts: E-Posters

### P01 A review of the changing use of cognitive assessment as part of the normal pressure hydrocephalus (NPH) diagnostic pathway

#### Cathryn Harries^1^, Aishah Hannan^1^, Samiul Muquit^2^, Samuel MT Jeffery^2^, Rupert Noad^1^

##### ^1^Department of Neuropsychology, Derriford Hospital, Plymouth, UK; ^2^South West Neurosurgery Centre, Derriford Hospital, Plymouth, UK

###### **Correspondence:** Cathryn Harries (cathryn.harries@nhs.net)

*Fluids Barriers CNS* 2021, **18(2)**: P01

**Introduction:** This study evaluates changes in the use of cognitive assessment in routine practice over 15 years in the NPH diagnostic pathway. In particular, it evaluates the impact of developing a joint multidisciplinary clinic, including Neuropsychology, and examines the impact on the demand for cognitive assessment.

**Methods:** Assessment and appointment data were collected via a retrospective review of electronic records of all patients with suspected NPH. Data collected included; cognitive tests undertaken, length of assessment and administrator qualifications. Data were collected at three time points spanning 15 years to allow a review of change in practice.

**Results:** A total of 148 patients (M = 91, F = 57) were seen. An increase in NPH activity was seen over the decade (T1 N = 30, T3 N = 88) with more patients having a formal cognitive assessment as part of their NPH evaluation upon commencement of the joint multidisciplinary clinic (T2 N = 10, T3 N = 49). Cost implications for an increased demand for Consultant Neuropsychologist time was mediated by a significantly increased Assistant Psychologist role.

**Conclusion:** The demand for neuropsychological involvement within the NPH pathway has increased over time. The main change has been in the proportion of patients having formal cognitive assessment as part of their evaluation. With the creation of an NPH multidisciplinary team patients are now routinely assessed at all stages of the pathway including at initial assessment, pre and post-surgical procedures and following shunt adjustment. We discuss the clinical capacity required to deliver this work ongoing and a model that is a cost-efficient use of the Consultant Neuropsychologist’s time.

### P02 Adult external hydrocephalus presenting as subdural haematoma following sinus venous thrombosis

#### L. Darie^1^, S.M. Toescu^1^, S. Khawari^1^, P. J. Grover^1^, A. K. Toma^1^

##### ^1^Department of Neurosurgery, National Hospital for Neurology and Neurosurgery, University College London Hospital, London, UK

###### **Correspondence:** Lucia Darie (darielucia@yahoo.com)

*Fluids Barriers CNS* 2021, **18(2)**: P02

**Introduction:** Hydrocephalus caused by venous sinus thrombosis in adults is exceedingly rare, with only a few existing case reports. Benign subdural frontal collections, as a form of external hydrocephalus, in infants can be associated with venous sinus stenosis. In adults, venous sinus thrombosis and bilateral hygromas can be seen in the context of intracranial hypotension and suspected cerebrospinal fluid leak.

**Case description:** A 49-year-old male with a background of metastatic prostate cancer was admitted following multiple falls whilst on anticoagulants (Tinzaparin) for pulmonary emboli. He complained of progressively worsening headaches, nausea and diplopia. The initial CT head showed bilateral subdural hygromas, which progressed after 8 days, with significant mass effect on the right hemisphere. He underwent burr hole drainage and evacuation of what appeared to be radiologically a right sided chronic subdural haematoma. Intraoperatively, the fluid appeared similar to CSF and was under high pressure. Fundoscopy revealed bilateral acute haemorrhagic papilloedema, and supranuclear vertical gaze palsy with Collier’s sign. Post-contrast MRI head and CT venogram revealed filling defects in the right transverse and sigmoid sinus as well as multiple stenoses and irregularities throughout the superior sagittal sinus, indicative of venous sinus thrombosis. The patient subsequently underwent image-guided insertion of ventriculo- peritoneal shunt. The CSF opening pressure was high.

**Conclusion**: We would like to point out the importance of the cerebral venous system in cerebrospinal fluid disturbances.

**Declarations**: The authors declare that the patient has give written consent for the publication of this study.

### P03 Assessment of thermal transcutaneous flow performance characteristics in asymptomatic patients with normal pressure hydrocephalus

#### Naomi Abel^1^, Konrad Bach^1^

##### ^1^Department of Neurosurgery and Brain Repair, University of South Florida, Tampa, FL, 33606, USA

###### **Correspondence:** Naomi Abel: (nabel@usf.edu)

*Fluids Barriers CNS* 2021, **18(2)**: P03

**Introduction:** Increased recognition of communicating or normal pressure hydrocephalus (NPH) in an aging population and technological improvements in ventriculoperitoneal shunts (VPS), specifically programmable valves, has resulted in an increase in diagnosis and treatment. Shunt malfunction is considered when a patient initially does not respond to VPS or has symptom recurrence. Malfunction may be evaluated with a shunt series for catheter disconnection or breaks and with a shunt patency study (SPS) for obstruction. SPS is invasive with risk of infection and incurs significant cost. Thermal transcutaneous flow (TTF) is a noninvasive alternative to evaluate for obstruction. While TTF is useful in evaluating flow in non- communicating hydrocephalus where there is high cerebrospinal fluid (CSF) pressure there is no data for communicating hydrocephalus with low or normal CSF pressure. This study assesses the performance characteristics of CSF flow using TTF in asymptomatic NPH (aNPH) .

**Methods:** 27 consecutive patients with aNPH and VPS at a single center prospectively underwent 2 tests in the sitting position. Specificity was calculated for one versus two tests.The manufacturer’s recommended threshold of 0.2 degree C temperature decrease was used to confirm flow.

**Results:** One test demonstrated 48 % and two tests demonstrated 59% specificity. Lowering the threshold to 0.15 increased the 2 test specificity to 78%.

**Conclusion:** TTF is a useful noninvasive screening tool to evaluate CSF flow in NPH;, however, cannot definitively rule in obstruction as there may be intermittent flow. 2 tests improve the specificity. Consideration is given to lowering the manufacture’s temperature threshold to confirm flow.

### P04 Cerebrospinal fluid biomarkers in idiopathic vs secondary normal pressure hydrocephalus

#### Andreas Eleftheriou*^1^, Johanna Rydja^2^, Zhang Yi^3^, Fredrik Lundin^1^

##### ^1^Department of Neurology and Department of Biomedical and Clinical Sciences, Linköping University, Linköping, Sweden; ^2^Department of Activity and Health and Department of Biomedical and Clinical Sciences, Linköping University, Linköping, Sweden; ^3^Department of Radiology, and Department of Health, Medicine and Caring Sciences, Linköping University, Linköping, Sweden

###### **Correspondence:** Andreas Eleftheriou (andelef2002@yahoo.gr)

*Fluids Barriers CNS* 2021, **18(2)**: P04

**Introduction:** Normal pressure hydrocephalus (NPH) is divided into an idiopathic and a secondary form. In a single-centre cohort we retrospectively aimed to analyse the cerebrospinal fluid (CSF) biomarkers in correlation with clinical data in each group.

**Methods:** The preoperative composition of t-tau, p-tau, β-amyloid, neurofilament (NFL) and glial fibrillary acidic protein (GFAP) in 88 shunted NPH-patients was analysed and correlated with motor functions [10-m-walk in steps (w10ms) and seconds (w10mt), timed-up and go test in seconds (TUGt) and steps (TUGs)] and Mini Mental State Examination (MMSE) before and 3 months postoperatively. 71 patients diagnosed with probable/possible iNPH (♂/♀:40/31, median age 75 years(y), range: 57-88y) and 17 patients with sNPH (♂/♀:12/5, median age 72y, range: 58-80y) were included from 1/1/2018 until 31/12/2019.

**Results:** Both iNPH- and sNPH-patients improved in gait postoperatively (p < 0.001, p = 0.003). β -amyloid was lower than normal (< 620ng/L) while t-tau (ref < 400ng/L) and p-tau (ref < 80ng/L) were normal in both groups. NFL was higher than normal in iNPH vs sNPH (2133 vs 1420). For iNPH (n = 58) NFL correlated with w10mt (r = 0.39, p = 0.002), w10ms (r = 0.37, p = 0.004), TUGt (r = 0.33, p = 0.01), TUGs (r = 0.37, p = 0.005), MMSE (r = -0.36 p = 0.005) and for t-tau and p-tau (r = 59); w10mt (r = 0.31, p = 0.01) and w10ms (r = 0.26, p = 0.04). In sNPH (n = 16) the only interesting correlations were with NFL and MMSE (r = -0.5, p = 0.05) and β-amyloid w10mt (r = -0.5, p = 0.049).

**Conclusion:** Low β-amyloid, normal t-tau and p-tau in both groups and elevated NFL in iNPH. Levels of the analysed biomarkers correlated moderately with symptoms.

### P05 Hemo and cerebrospinal fluid dynamics in premature newborn with intraventricular hemorrhage

#### B De Waele^1^, M Aye^2^, C CapeI^1,3^, C Gondry jouet^1,2^, O Baledent^1,4^

##### ^1^Chimere UR 7516, University of Picardie Jules Verne, Amiens, France; ^2^Radiology department, University hospital, Amiens, France; ^3^Neurosurgery department, University hospital, Amiens, France; ^4^Image processing department, University hospital, Amiens, France.

###### **Correspondence:** Baptiste De Waele (baptiste.dewaele60290@laposte.net)

*Fluids Barriers CNS* 2021, **18(2)**: P05

**Introduction :** Premature newborn lntraventricular hemorrhages (IVH) is often associated with ventricular dilatation. Classical MRI protocol investigate morphology of the brain without CSF and cerebral blood flows (CBF) quantification. Phase contrast MRI (PC-MRI) can quantify CSF and (CBF) in adults. The objective was to quantify potential alterations of these cerebral flows in IVH newborns.

**Material and methods:** 12 premature newborn between 22 and 39 weeks whom presented active IVH were investigated by a 1.5 T MRI. PC-MRI acquisitions were added to the morphological acquisition to quantify CSF and CBF. These flows dynamics curves were reconstructed during the cardiac cycle to calculate CSF and blood volume displacement through the cranio spinal compartments.

**Results:** CBF was well correlated with mass of the patients (R^2^ = 0. 87; p < 10^-6^) and also between intracranial and cervical level (R^2^ = 0.79; p = 0.0.37). In response of intracranial blood volume expansion during cardiac cycle CSF flush in the spinal canal was very small in 8 subjects and hyper dynamic in one. The CSF flow in the aqueduct was null in 4; pseudo-normal in 6 and hyper dynamics in 2 patients.

**Conclusion:** PCMRI can quantify CBF and CSF in newborn to show that IVH not only impact the brain morphology but also CSF dynamics at different locations of the cranio spinal compartments. Impact can result as a blockage or by an hyper dynamic CSF flow. PC MRI bring complementary informations to the morphological analysis that could be helpful for the understanding of physiopathology and in the different surgical technics to treat hydrocephalus.

### P06 Intracranial pressure is affected by sedation in the first 24 hours of monitoring

#### Melissa De Gouveia^1^, Linda D’Antona^1^, Lewis Thorne^1^, Laurence Watkins^1^, Ahmed Toma^1^

##### ^1^Department Of Neurosurgery, National Hospital For Neurology And Neurosurgery; London

###### **Correspondence:** Melissa De Gouveia (melgouv@hotmail.com)

*Fluids Barriers CNS* 2021, **18(2)**: P06

**Introduction:** Intracranial pressure (ICP) monitoring is a valuable tool to diagnose and treat disorders of cerebrospinal fluid (CSF) dynamics, hydrocephalus and head injuries. ICP monitoring probes can be inserted with local anaesthetics or/and sedation. The reliability of ICP measurements taken under the effect of sedative agents is unclear.

**Methods:** A retrospective study includes patients who underwent 48 hours of ICP monitoring with ICP probes inserted with local anaesthetic or sedation to investigate CSF dynamics disturbances. Local anaesthetic used was 1% lignocaine. General anaesthetic ranged from midazolam, propofol or fentanyl. The median 24-hour ICP results of the first and second day of ICP monitoring were compared (Wilcoxon signed-rank test). The comparison was stratified by type of anaesthetic (local versus sedation).

**Results:** Twenty patients undergoing 48-hour ICP monitoring were identified (14 females, mean age 42 years). Ten patients had ICP probes inserted with local anaesthetic and 10 with sedation. The baseline characteristics of the two groups were similar. The mean difference between the second day of ICP data recording and the first day was -1.7 (3.6 SD) mmHg for the local anaesthetic and +1.9 (3.1 SD) in the sedation group, and the difference between these two means was statistically significant (Wilcoxon signed-rank test p = 0.019).

**Conclusion:** The results of this study suggest that sedation may affect ICP measurements; this factor should be taken into account when interpreting the results of ICP monitoring. More extensive studies will be needed to confirm this finding and investigate the duration of sedation on ICP monitoring results and the effect of different anaesthetic agents.

### P07 Overdrainage in an isolated fourth ventricle

#### Borja Sanz Peña MD.^1^, Irene Iglesias Lozano Phd.^1^, Sanriago Rocha Romero MSc-MD.^1^

##### ^1^Deparment of neurosurgery, Hospital Universitario Puerta del Mar, Cádiz, Spain.

###### **Correspondence:** Borja Sainz Pena (bsanzpena@gmail.com)

*Fluids Barriers CNS* 2021, **18(2)**: P07

**Introduction:** Isolated fourth ventricle is a rare and late complication insertion of lateral ventricular shunt for hydrocephalus in children and adults. A history of prematurity, hydrocephalus secondary to intraventricular hemorrhage or infection is common in this rare entity. Isolated fourth ventricle is reported at around 2.5% of some series, with an interval from ventriculoperitoneal shunt (VPS) insertion of 1–7 years.

**Methods:** Treatment strategies for this pathology go from the colocation of a free tube or connected to a valve and endoscopic management with aqueductoplasty with or without stenting of the Sylvius aqueduct. Previously reported complications with the shunting of the fourth ventricle are cranial nerve palsies and injuries to the floor of the fourth ventricle. Here we present a case of a 6 years old girl with a post hemorrhagic hydrocephalus with a VPS that went through a suboccipital endoscopic aqueductoplasty with stent of an isolated fourth ventricle. Because of neurological deterioration and increased size of the isolated ventricle, she required a free tube to the fourth ventricle. Control magnetic resonances showed a normal size of the fourth ventricle and a transtentorial herniation of the occipital horn of the left lateral ventricle and a thinning of the pons.

**Results conclusion:** After the implantation of an adjustable valve, the transtentorial herniation, the slendering of the pons and the neurological status improved but the size of the fourth increased to nearly normal size.

### P08 Referred shoulder tip pain from lumbo-peritoneal shunt’s distal catheter; under-reported neurosurgical presentation

#### Oyeleye Egunjobi^1^

#### ^1^Department of Neurosurgery, University Hospitals Plymouth, United Kingdom.

##### **Correspondence:** Oyeleye Egunjobi (leyemmanuel@yahoo.com)

*Fluids Barriers CNS* 2021, **18(2)**: P08

**Introduction:** Hydrocephalus is a neurological condition which entails cerebrospinal fluid build-up in the CSF space. In management of hydrocephalus, diversion systems are often used called shunts.

**Case report:** 20 year old lady with hydrocephalus, latest clinical procedure was insertion of a lumbo-peritoneal shunt (LPS). She presented with two week history of abdominal pain, radiating towards the neck and tip of left shoulder, associated with constipation. Examination revealed tenderness in left hypochondriac region, no surgical site leaks nor discharge. Neurologically, Glasgow Coma Score was 15/15, normal visual findings. Fluoroscopy revealed dilated loops of bowel compressing distal catheter against peritoneum, abdominopelvic ultrasound, ECG, CRP and urinalysis were normal, Beta hCG was negative. Diagnosis of referred pain secondary to distal shunt catheter peritoneal irritation was made. Patient was conservatively managed, improved and discharged.

**Discussion:** Few literatures have reported referred pain as complication of peritoneal shunts; similarly this case presents and explores the need for more reported cases if noted. It can be seen that it’s still important to rule out possible red flag diagnoses as potential differentials. General surgical input and advice was required in this case, of which recommendation was conservative management of constipation being culprit of distended bowel loops, mechanically pressing against distal catheter of the shunt.

**Conclusion:** Referred pain in peritoneal shunts is a rare complication or perhaps under- reported, therefore necessary to be includmyped when obtaining consents from patients ahead of shunting procedures. This case emphasizes need for consideration of referred shoulder tip pain as recognized complication of ventriculo-peritoneal or Lumbo-peritoneal shunts.

### P09 The variation of optic nerve sheath diameter (ONSD) measured by ultrasound before and after the surgery for hydrocephalus

#### Mindaugas Urbonas^1,2^, Algimantas Matukevicius^1^, Arimantas Tamasauskas^1,2^, Vytenis Pranas Deltuva^1,2^, Adomas Bunevicius^2^

##### ^1^Department of Neurosurgery, The Hospital of Lithuanian University of Health Sciences (LSMU) Kauno klinikos; ^2^Neuroscience Institute, Lithuanian University of Health Sciences

###### **Correspondence:** Urbonas Mindaugas (mindaugas.urbonas@kaunoklinikos.lt)

*Fluids Barriers CNS* 2021, **18(2)**: P09

**Objectives/aim:** Little is known about the value of the change in optic nerve sheath diameter (ONSD) during the treatment and follow up of hydrocephalus. The aim of this report was to investigate the variation of ONSD measured by ultrasound before the surgical treatment of hydrocephalus and 4 – 5 days after the operation.

**Methods:** 20 adult patients (mean age 56.8 ± 15.98 (SD) years) were operated for hydrocephalus (7 endoscopic third ventriculostomies and 13 ventriculoperitoneal shunt insertions). ONSD was measured 3 mm behind the papilla in each eye by ultrasound in two positions—standing and supine. The variation of ONSD was calculated as follows: ((ONSD supine position–ONSD standing position)/ONSD supine position) x 100%. Also MRI scans were done for the patients before and 4–5 days after the operation.

**Results:** Preoperatively, ONSD variation was 3.74% in the right eye and 5.7% in the left eye. 4–5 days after operation, ONSD variation was 5.74% in the right eye and 6.88% in the left eye. There was a strong correlation between ONSD in supine position measured by ultrasound and ONSD measured from MRI scans (preoperatively and after the operation).

**Conclusion:** We observed that ONSD measured by ultrasound changes significantly after the surgical treatment for hydrocephalus. We propose that lower intracranial pressure after the operation could be related with the greater narrowing of ONSD especially in standing position. The variation of ONSD is directly related with the changes of intracranial pressure and could be used for the evaluation and follow up of the patients with hydrocepalus. Larger studies, in a wider ranging population, are required to establish how widely these data apply.

### P10 Vascular stiffness in normal pressure hydrocephalus

#### Ian McKnight^1^, Regan Raines^1^, Hunter White^1^, In-Hyun Park^2^, Joon W. Shim^1^

##### ^1^Department of Biomedical Engineering, Marshall University, USA; ^2^Department of Genetics, Yale University School of Medicine, USA.

###### **Correspondence:** Joon Shim (shim@marshall.edu)

*Fluids Barriers CNS* 2021, **18(2)**: P010

**Introduction:** The development of vascular stiffening (VS) is a risk factor of normal pressure hydrocephalus (NPH). The mechanism of VS remains ambiguous, and there is currently no prevention for this pathological condition.

**Methods:** Here, we studied whether phospholipase A2 group 6 (PLA2g6)/Ca^2+^ signaling was involved in arterial stiffness induced by aging. Aortic stiffness was measured *in vivo* by pulse wave velocity (PWV) in wild type (WT) mice and animals in which PLA2g6/Ca^2+^ function was constitutively impaired.

**Results:** We found that aging significantly increased PWV in WT, but not in PLA2g6ex2^KO^ mice. *In vitro* analysis of vascular smooth muscle cells (SMCs) from PLA2g6ex2^KO^ animals showed a significant impairment of PLA2g6-dependent Ca^2+^ signaling and reduced proliferation. Histology revealed that the thickness of aortic media was decreased in aged PLA2g6ex2^KO^ mice. Analyses of SMC layer of thoracic aorta from the aged (24 months old) mice revealed a significant elevation of BCL11B whose deficiency is known to stiffen arteries. Furthermore, RT-PCR of human postmortem caudate nucleus of patients with NPH showed differential gene expressions of transmembrane proteins (TMEMs), which mediate hydrocephalus.

**Conclusion:** Our results demonstrate that aortic stiffening caused by aging can be prevented by inhibition of PLA2g6/Ca^2+^ signaling. This effect is associated with reduced SMC proliferation and increased BCL11B in the vasculature. Discovery of the previously unknown role of PLA2g6/Ca^2+^ in age-related VS revealed a new molecular mechanism that can promote BCL11B in aged individuals and may offer a novel target for prevention of pathological VS and NPH.

